# Supplementary material for: Self-assembled three dimensional network designs for soft electronics
Source: Nat Commun. 2017 Jun 21;8:15894. doi: 10.1038/ncomms15894 (PMC5482057; doi:10.1038/ncomms15894)
Supplement: Supplementary Information [file ncomms15894-s1.pdf]

Type of file: PDF
Size of file: 0 KB
Title of file for HTML: Supplementary Information
Description: Supplementary Figures and Supplementary Discussion

Type of file: AVI
Size of file: 0 KB
Title of file for HTML: Supplementary Movie 1
Description: Device demonstration with an inductive power transmitter and a cell phone with customized application: The device continuously monitors 3 axis acceleration (related with respiration rate) and electrophysiological activity from the skin of the chest and transmit health information, which is extracted from raw data, via Bluetooth protocol.

Type of file: PDF
Size of file: 0 KB
Title of file for HTML: Peer Review File
Description:

Supplementary Discussion: Discussions on the scheme of two-stage encapsulation

The scheme of two-stage encapsulation can yield significant improvements in the elastic stretchability, compared to the usual method of introducing the encapsulation at the final stage of fabrication and assembly. For the device layout in Fig. 2a ($E_{substrate}$ = 20 kPa and $E_{encapsulation}$ = 3 kPa), considerable enhancements in elastic stretchability (120% vs 50% for X-axis, 123% vs 51% for Y-axis, and 84% vs 26% for radial stretching, respectively) can be achieved for an encapsulation strain (ε_{encap}) close to 39%. In particular, the elastic stretchability for uniaxial stretching approaches that of the unencapsulated counterpart (e.g., 120% vs 144% for X-axis and 123% vs 146% for Y-axis). The radial stretchability (~ 84%) in this condition is, however, smaller than the uniaxial stretchabilities (120% for X-axis and 123% for Y-axis), due to the different degrees of mechanical interaction between encapsulation and 3D coils. Specifically, the encapsulation material experiences more significant deformations under radial than uniaxial stretching, thereby leading to a stronger mechanical interaction with 3D coils. Taking 50% uniaxial stretching as an example, the Poisson effect induces only ~18% compressive strain in the encapsulation along the thickness (i.e., out-of-plane) direction. For 50% radial stretching, the Poisson effect induces ~ 56% compressive strain in the encapsulation along the thickness direction, which is much higher than uniaxial stretching. The different levels of compressive strain in encapsulation then induce different levels of strain increase in the 3D coils via the mechanical interaction.

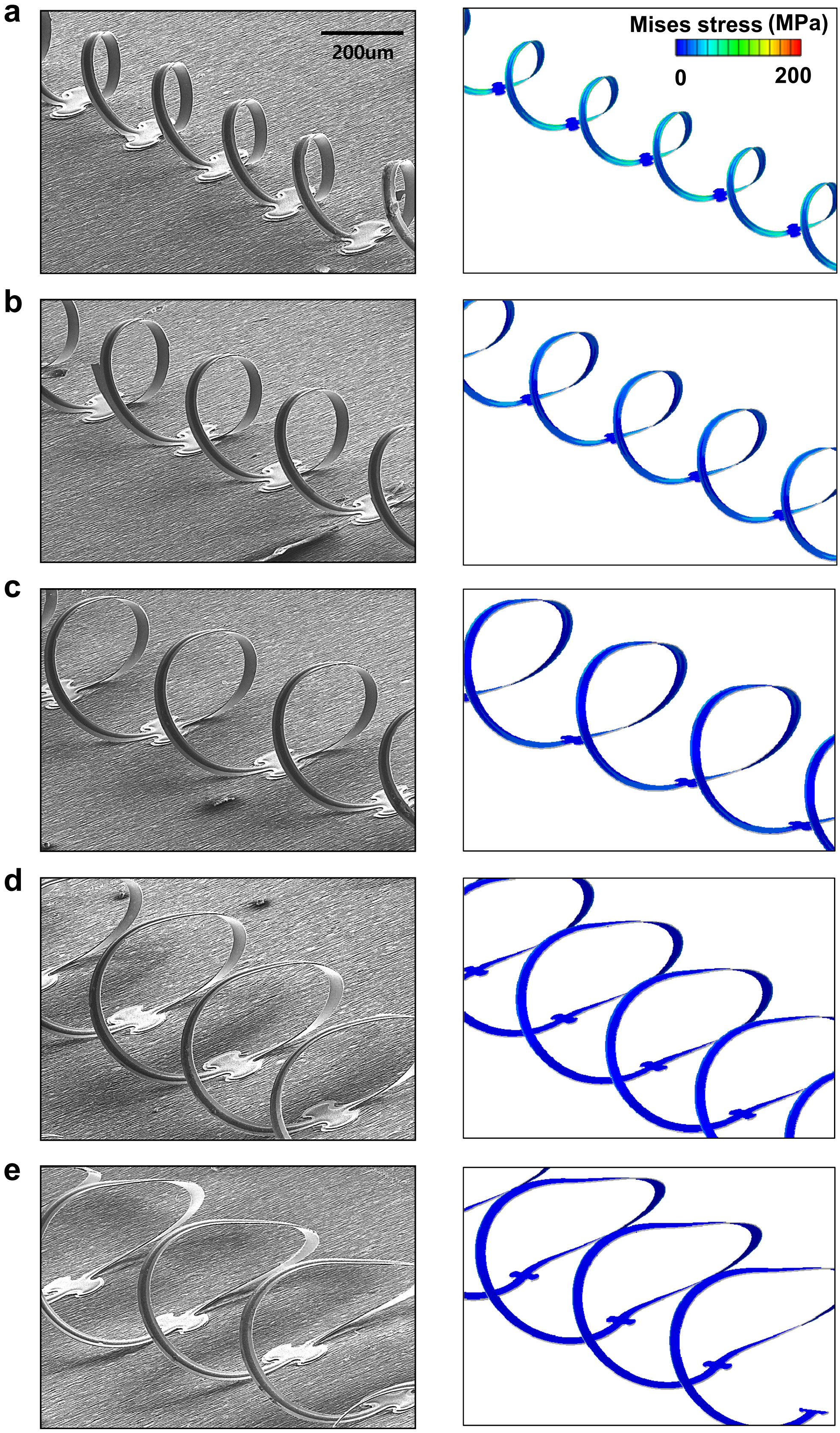

Supplementary Figure 1. SEM images and corresponding FEA results for 3D helical interconnects formed from 2D serpentine microstructures with different arc angles: (a) 120°, (b) 150°, (c) 180°, (d) 210° and (e) 240°.

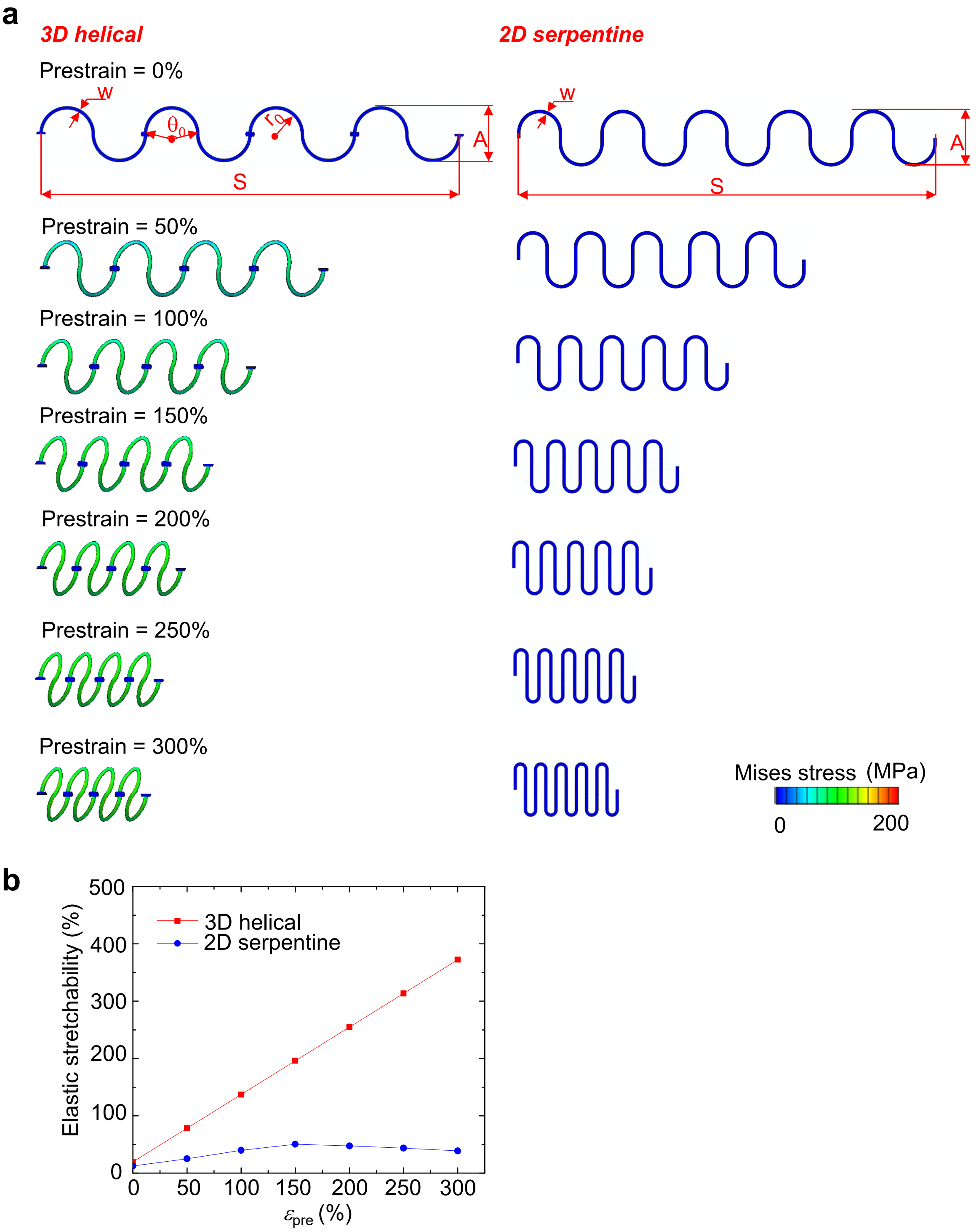

Supplementary Figure 2. Schematic illustration of configuration for 3D helical and 2D serpentine interconnects and results of elastic stretchability. (**a**) Configuration and Mises stress distribution of 3D helical interconnect and 2D serpentine interconnect, with prestrain 0% (precursor), 50%, 100%, 150%, 200%, 250%, 300%, respectively. Illustration of geometric parameters is shown in 2D precursor (prestrain=0%). (**b**) Elastic stretchability of the 3D helical coils in (**a**) and 2D serpentine with similar geometric parameters as a function of prestrain used in the assembly.

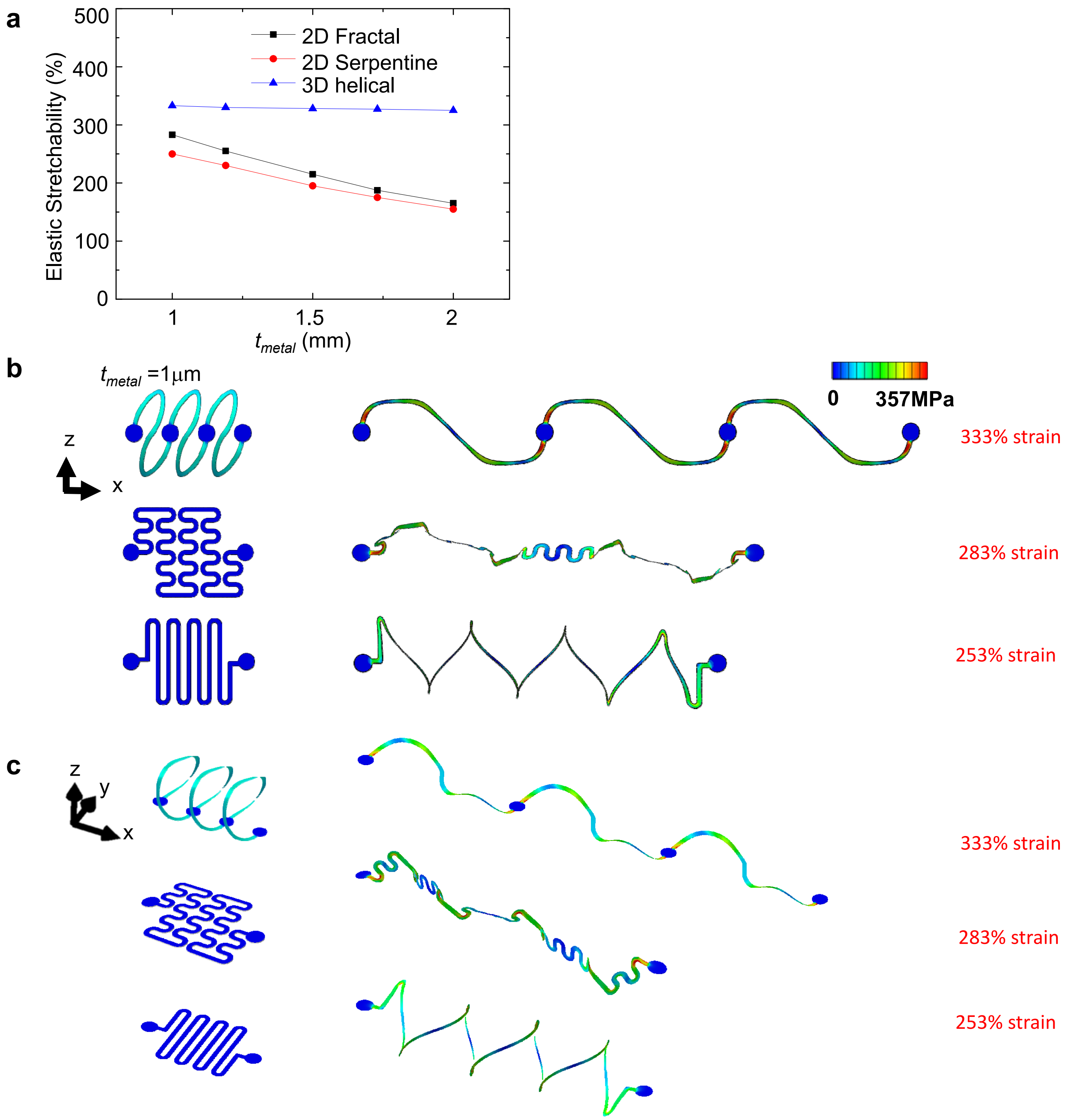

Supplementary Figure 3. Mechanics of 3D helical interconnects in comparison to end-bonded 2D serpentine and fractal interconnects in the unencapsulated condition. (**a**) Elastic stretchability versus metal (Cu) thickness (t_{metal}) in the unencapsulated condition, for 2D serpentine, 2D fractal and 3D helical interconnects. For the purpose of comparison, the key geometric parameters (width, thickness, span, and amplitude) are approximately the same for the three interconnects. The interconnects are all made of single-layer copper. (**b**) FEA results on the configurations of 3D helical, 2D fractal and serpentine interconnects before and after stretched to the corresponding elastic limit, from a top-view perspective. (**c**) Similar results from a 3D-view perspective. The color represents the magnitude of Mises stress.

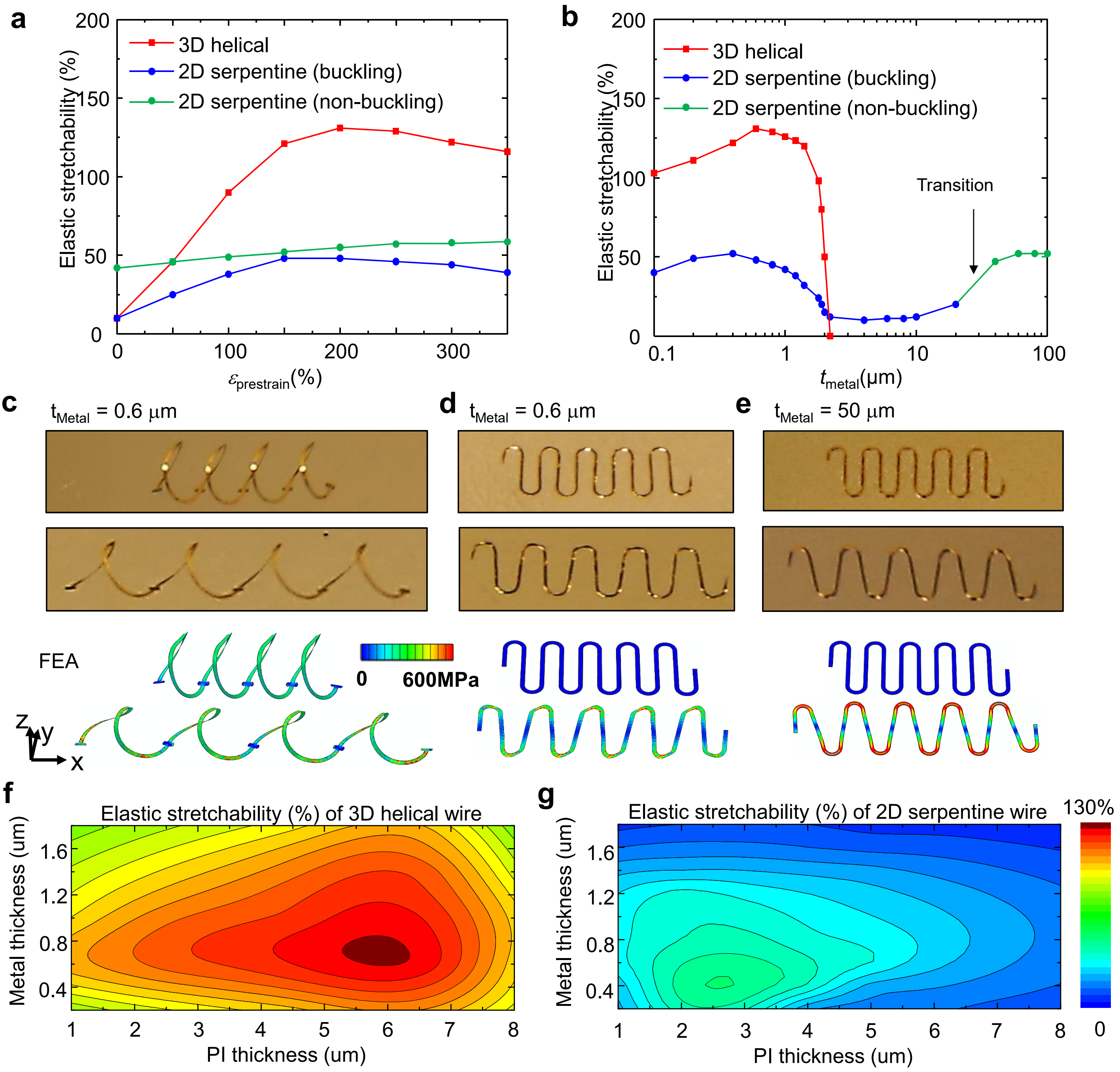

Supplementary Figure 4. Mechanics of 3D helical interconnects in comparison to fully-bonded 2D serpentine interconnects in the encapsulated condition. (**a**) Elastic stretchability versus the prestrain of substrate, for the 3D helical and 2D serpentine interconnects encapsulated by a low-modulus (~ 3 kPa) silicone. The metal (Ni) thickness is t_{metal}=0.6 μm for both the helical interconnects and the 2D serpentine interconnects with local wrinkling, and t_{metal}=50 μm for the 2D serpentine interconnect with global buckling. (**b**) Elastic stretchability of helical and serpentine interconnects versus the metal thickness for a fixed prestrain (ε_{pre}=200%). (**c** - **e**) Optical images and FEA predictions on the un-stretched and stretched configurations of three interconnects, including a helical interconnect with t_{metal}=0.6 μm and $\varepsilon_{prestrain}$=200%, and two serpentine interconnects with t_{metal}=0.6 μm and 50 μm. (**f**) Contour graph of elastic stretchability among various thicknesses of metal and PI layers for the 3D helical interconnects. (**g**) Similar results for the 2D serpentine interconnects.

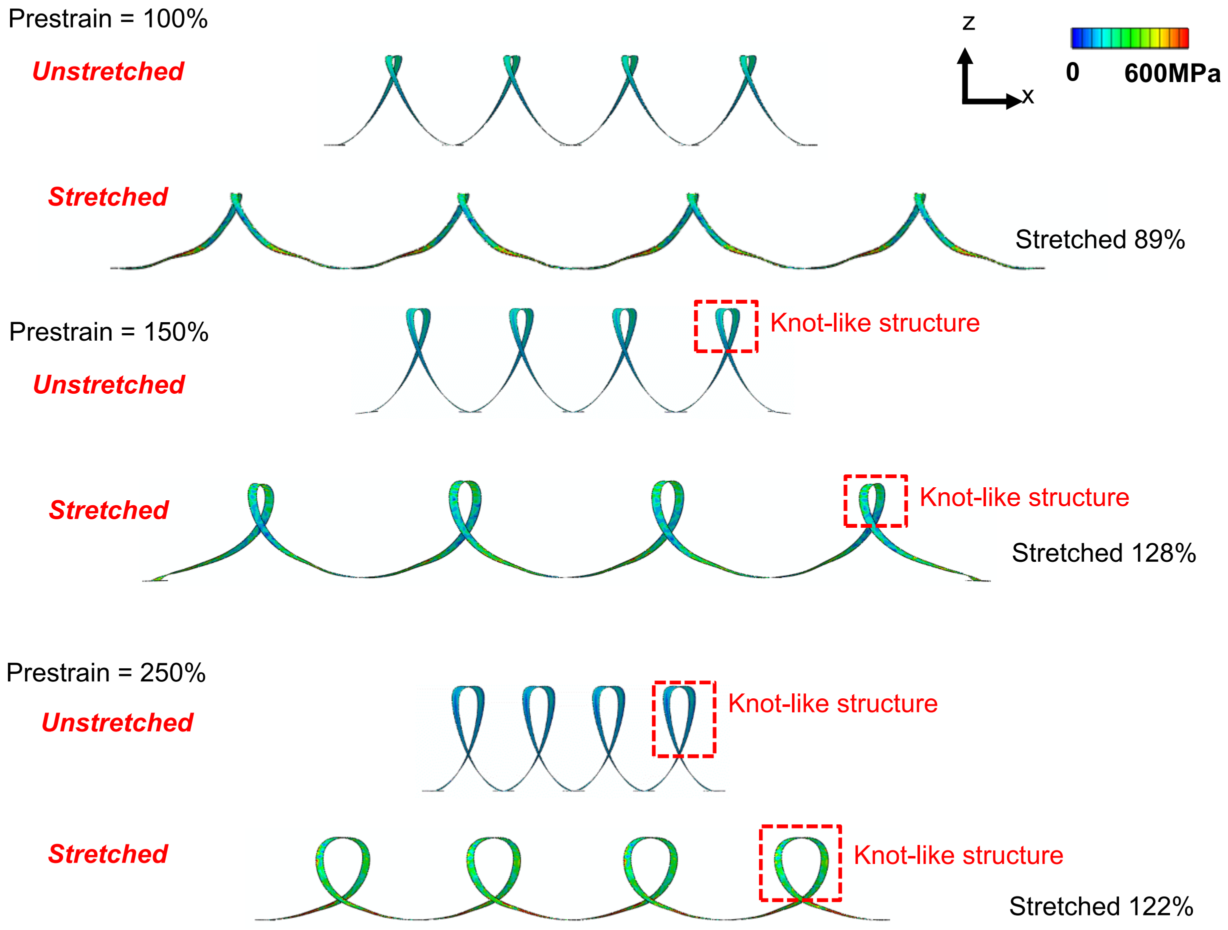

Supplementary Figure 5. Effect of prestrain on the deformations of encapsulated 3D helical interconnects. Deformed configurations of three helical interconnects (Ni, t_{metal}=0.6 µm, t_{PI}=6.0 µm, $E_{encapsulation}$=3 kPa, $E_{substrate}$=20 kPa) formed with different prestrains (100%, 150% and 250%), when stretched to the corresponding elastic limit. The color represents the magnitude of Mises stress.

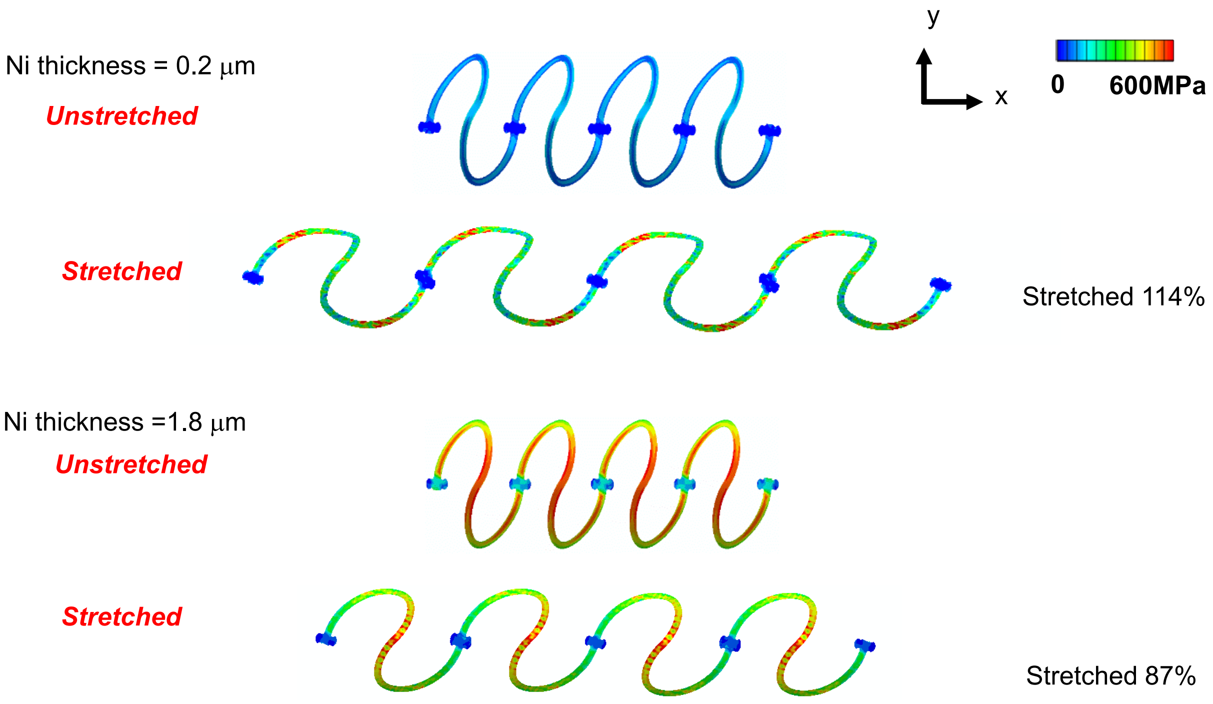

Supplementary Figure 6. Effect of metal thickness on the Mises-stress distribution of encapsulated 3D helical interconnects. Deformed configurations of helical interconnects (Ni, ε_{pre}=200%, $E_{encapsulation}$=3kPa, $E_{substrate}$=20kPa) with two different thicknesses (0.2 μm and 1.8 μm), when stretched to the corresponding elastic limit. The color represents the magnitude of Mises stress.

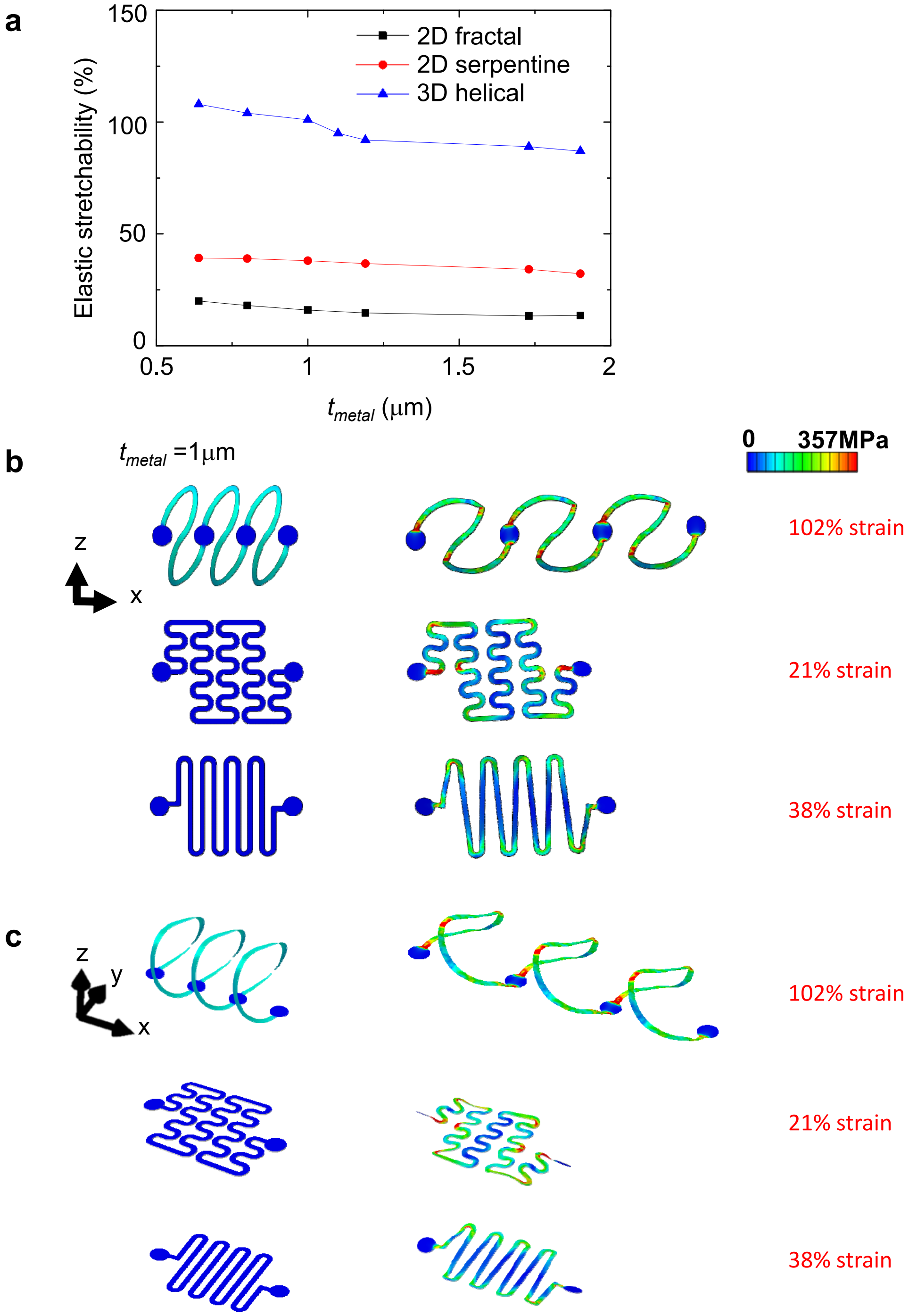

Supplementary Figure 7. Mechanics of 3D helical interconnects in comparison to fully-bonded 2D serpentine and fractal interconnects in the encapsulated condition. (**a**) Elastic stretchability versus metal (Cu) thickness (t_{metal}) in the encapsulated condition ($E_{encapsulation}$=3 kPa), for 2D serpentine, 2D fractal and 3D helical interconnects. For the purpose of comparison, the key geometric parameters (width, thickness, span, and amplitude) are approximately the same for the three interconnects. (**b**) FEA results on the configurations of 3D helical, 2D fractal and serpentine interconnects before and after stretched to the corresponding elastic limit, from a top-view perspective. (**c**) Similar results from a 3D-view perspective. The color represents the magnitude of Mises stress in the metal layer.

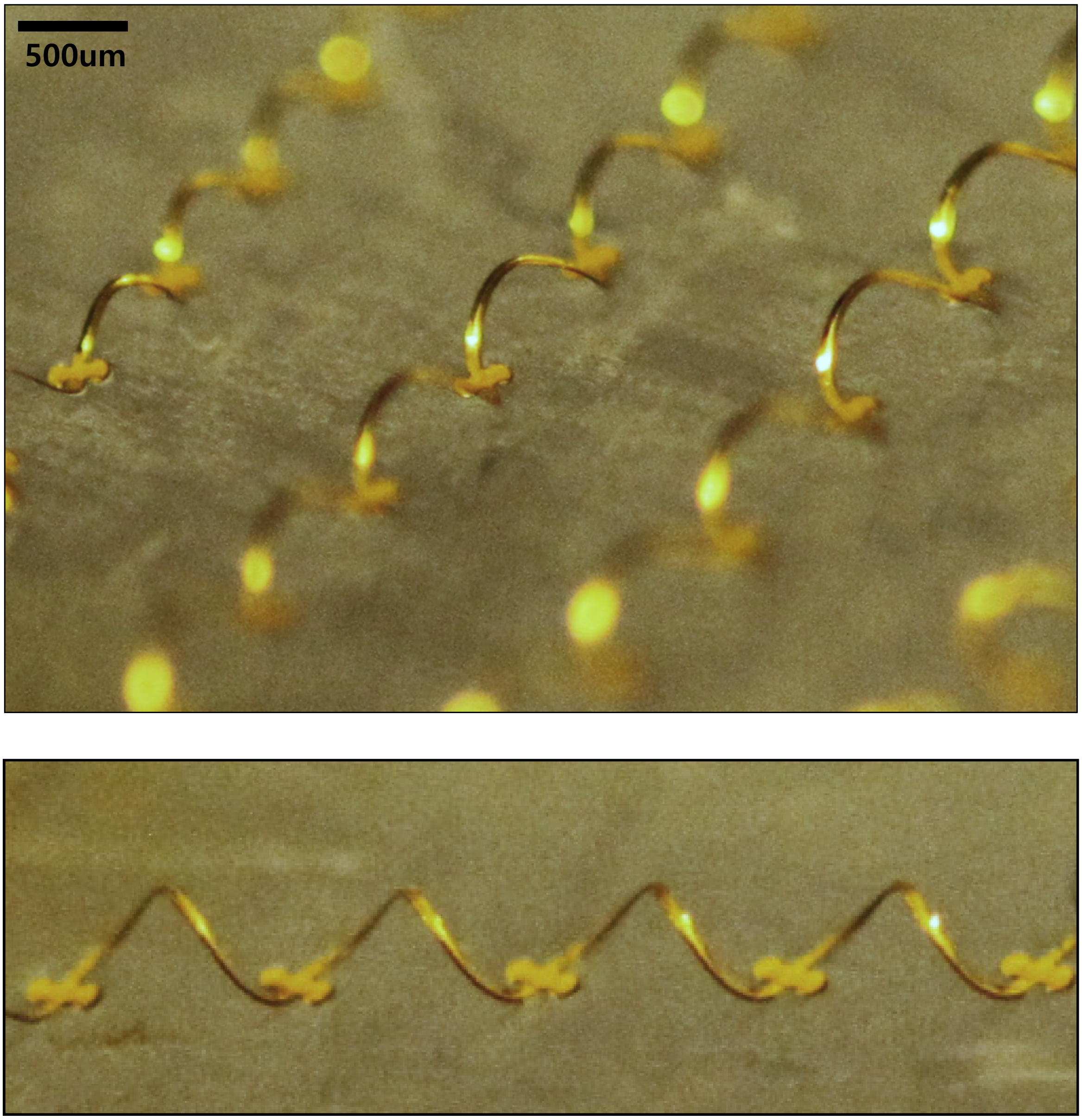

Supplementary Figure 8. Fabricated 3D coil array

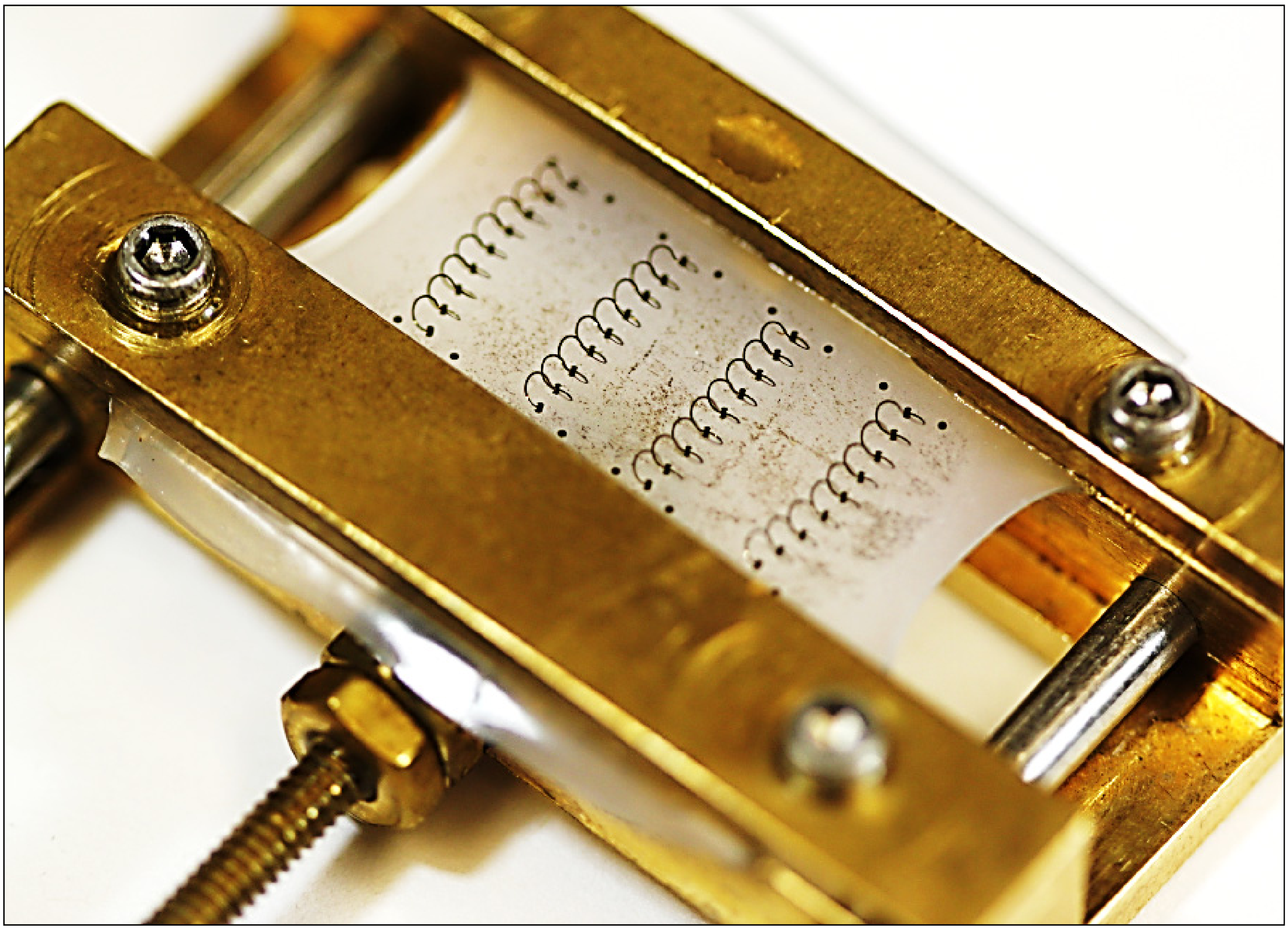

Supplementary Figure 9. Fabricated 3D coil array

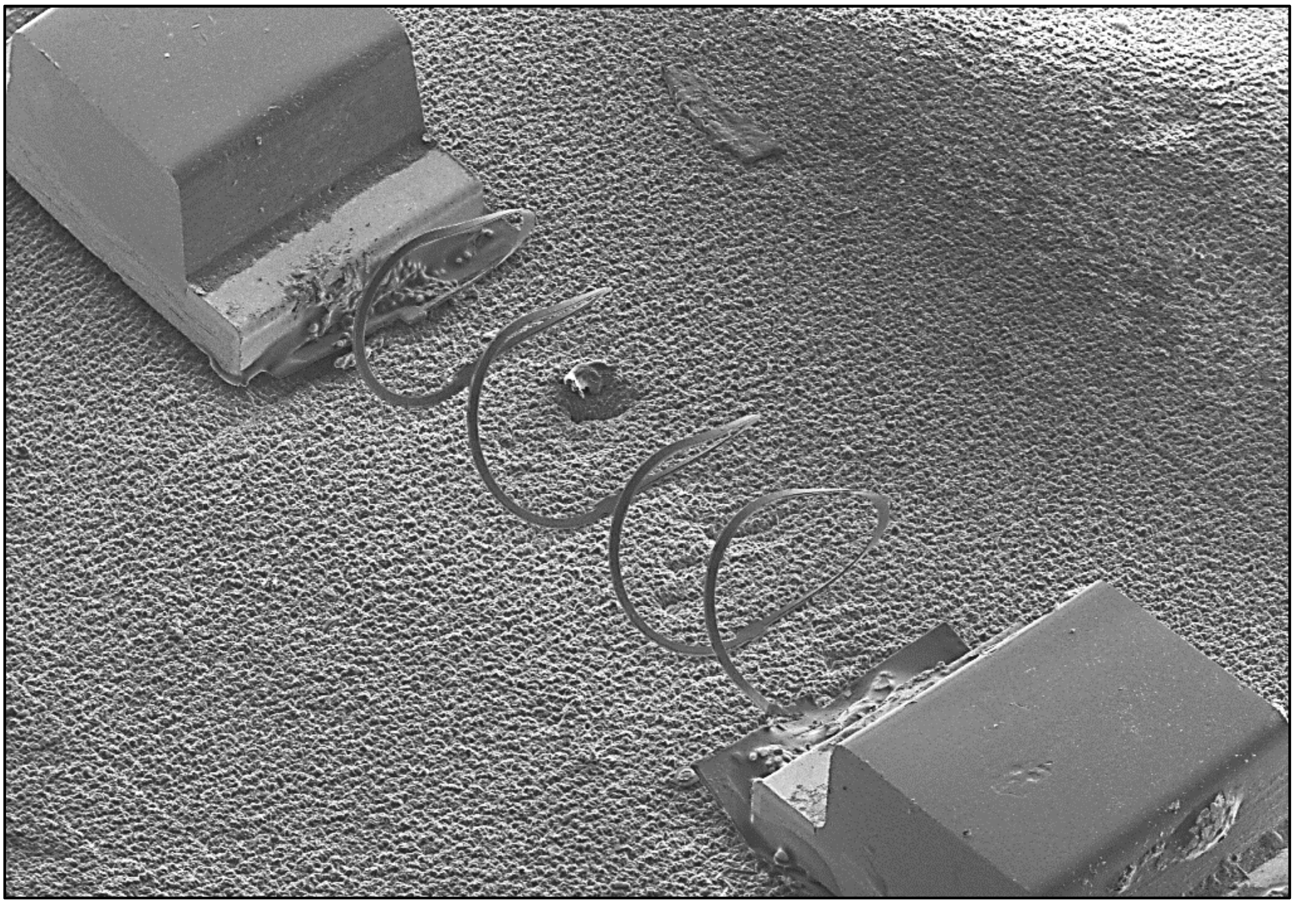

Supplementary Figure 10. Commercial LED chips connected by 3D conductive coils

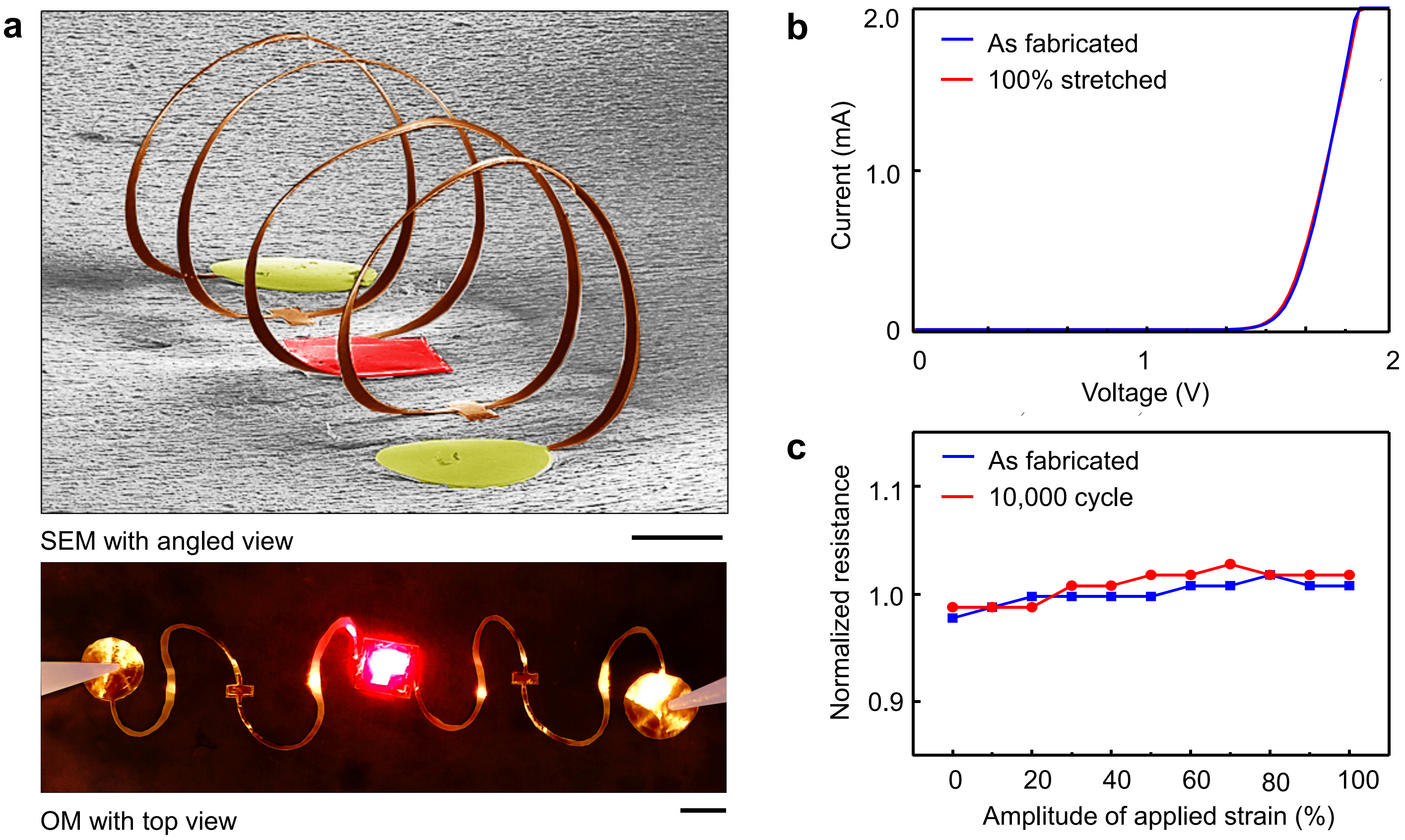

Supplementary Figure 11. Thin film InGaAs LED components connected by 3D conductive coils: (a) SEM and OM images of the fabricated stretchable LED system with coil, (b) I-V curve of the LED system, (c) Mechanical fatigue test up to 10,000cycles

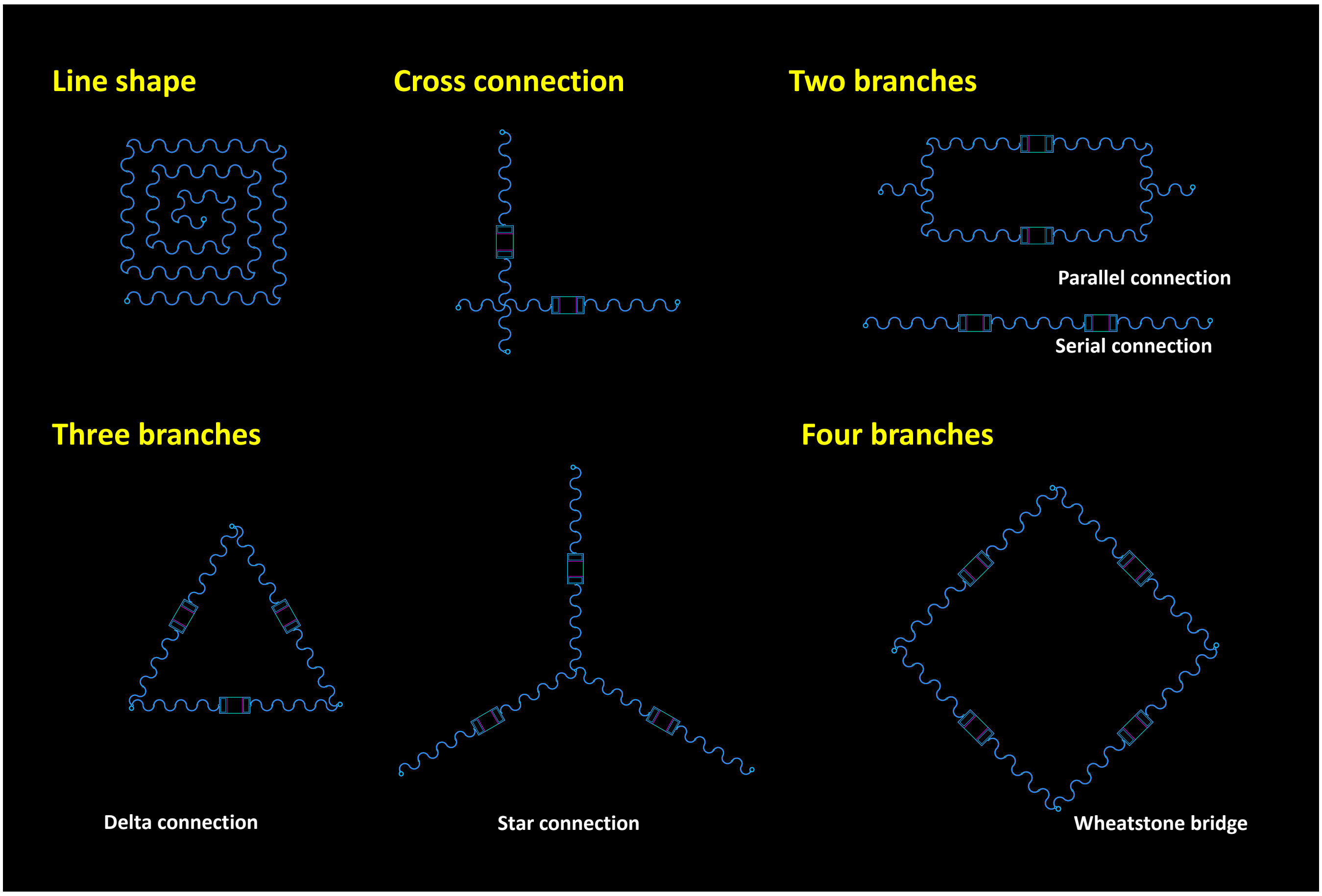

Supplementary Figure 12. Mask design of 3D coil fabrication for basic element of electrical topology

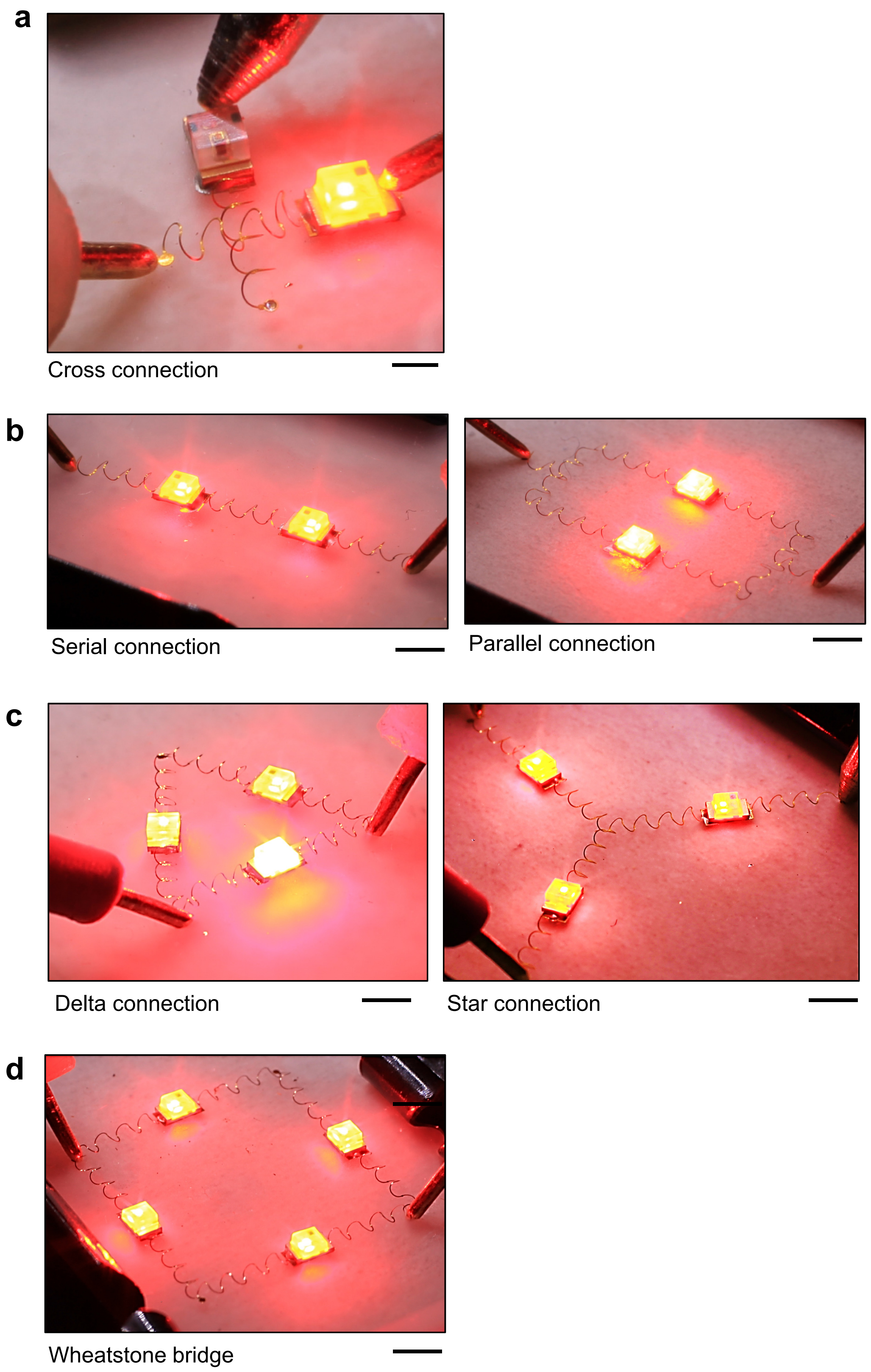

Supplementary Figure 13. 3D coil fabrication for basic element of electrical topology Scale bar is 3 mm.

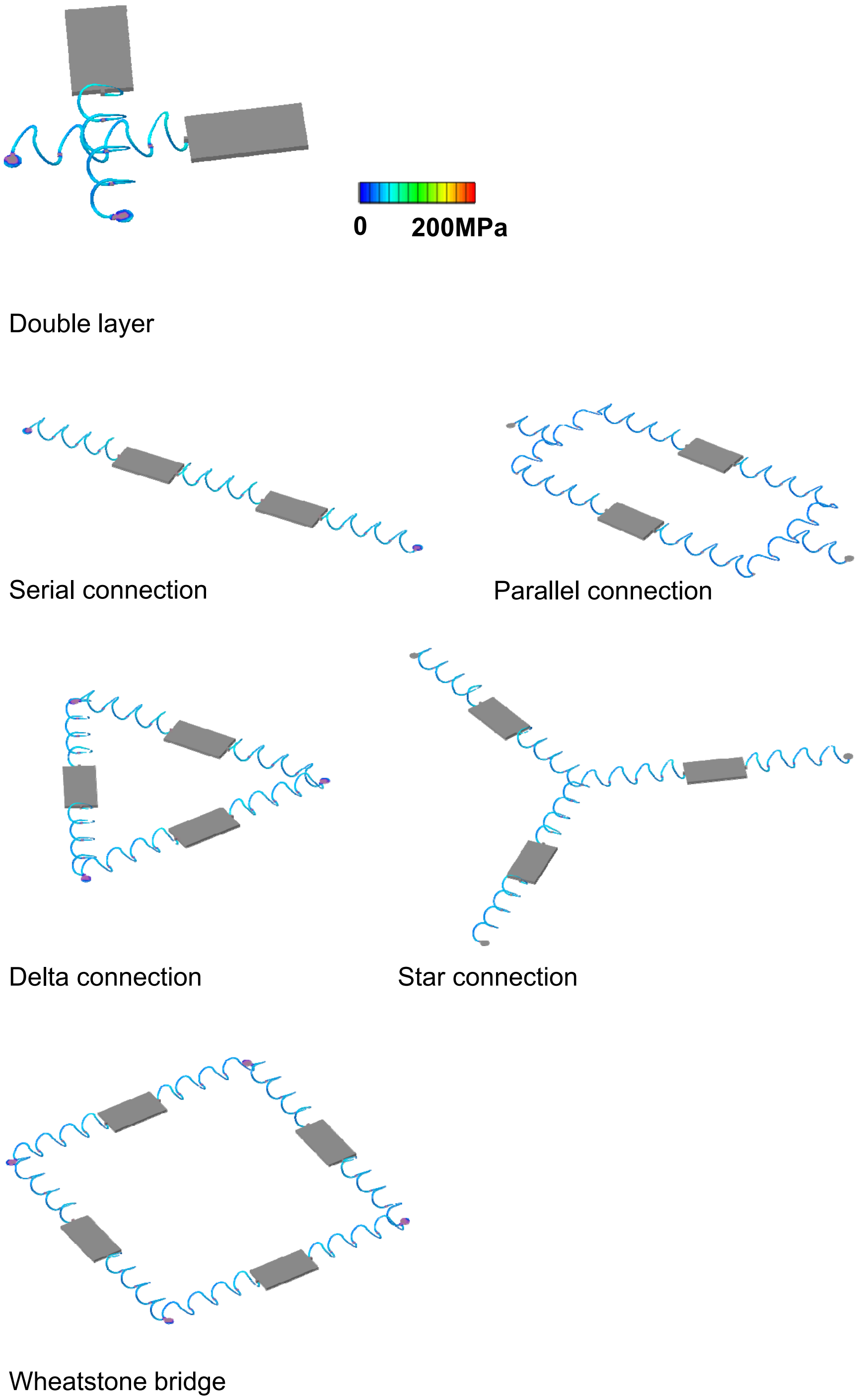

Supplementary Figure 14. FEA predictions on the 3D configurations of basic elements of electrical topology shown in Supplementary Figure 13.

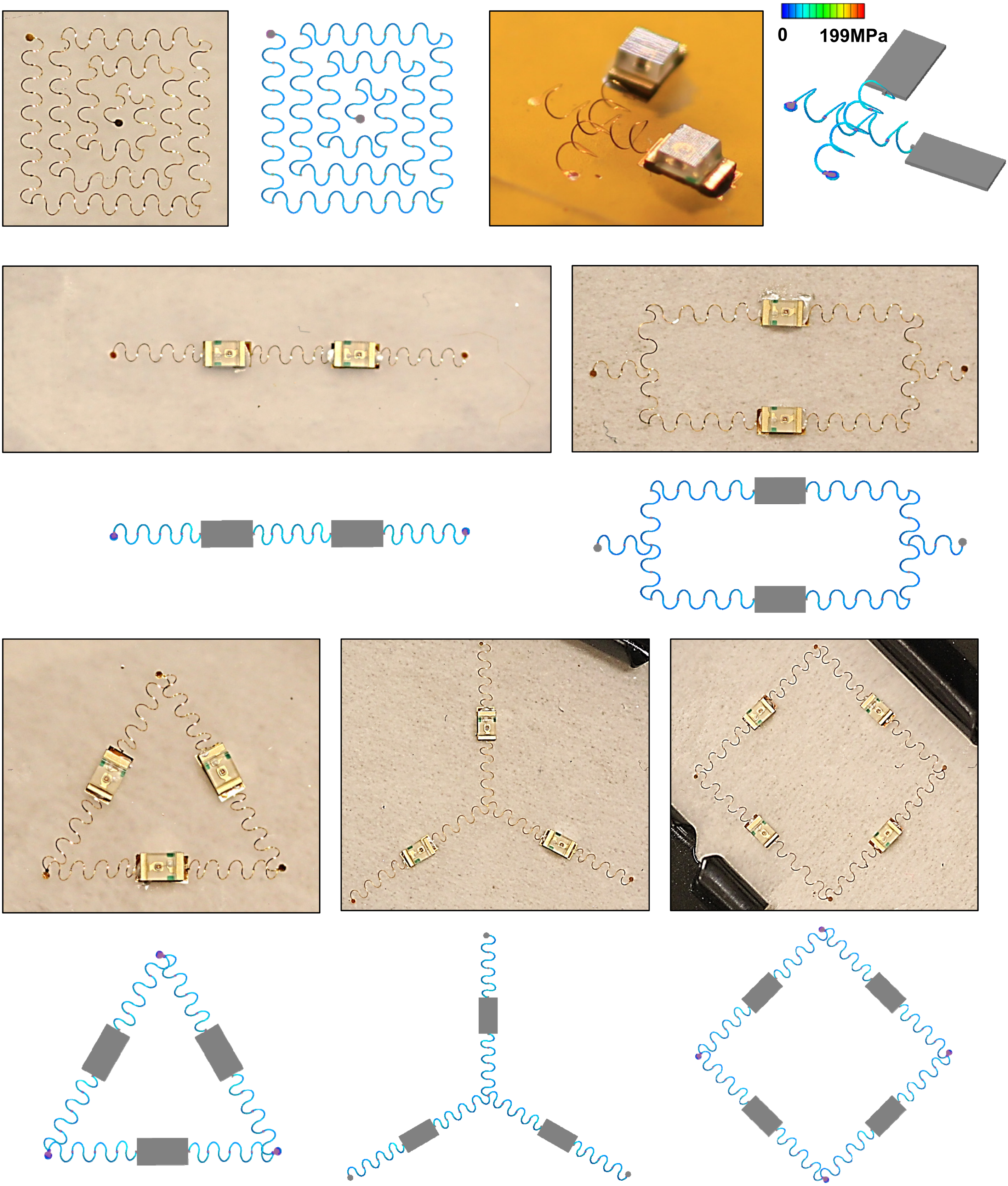

Supplementary Figure 15. FEA results for basic circuits

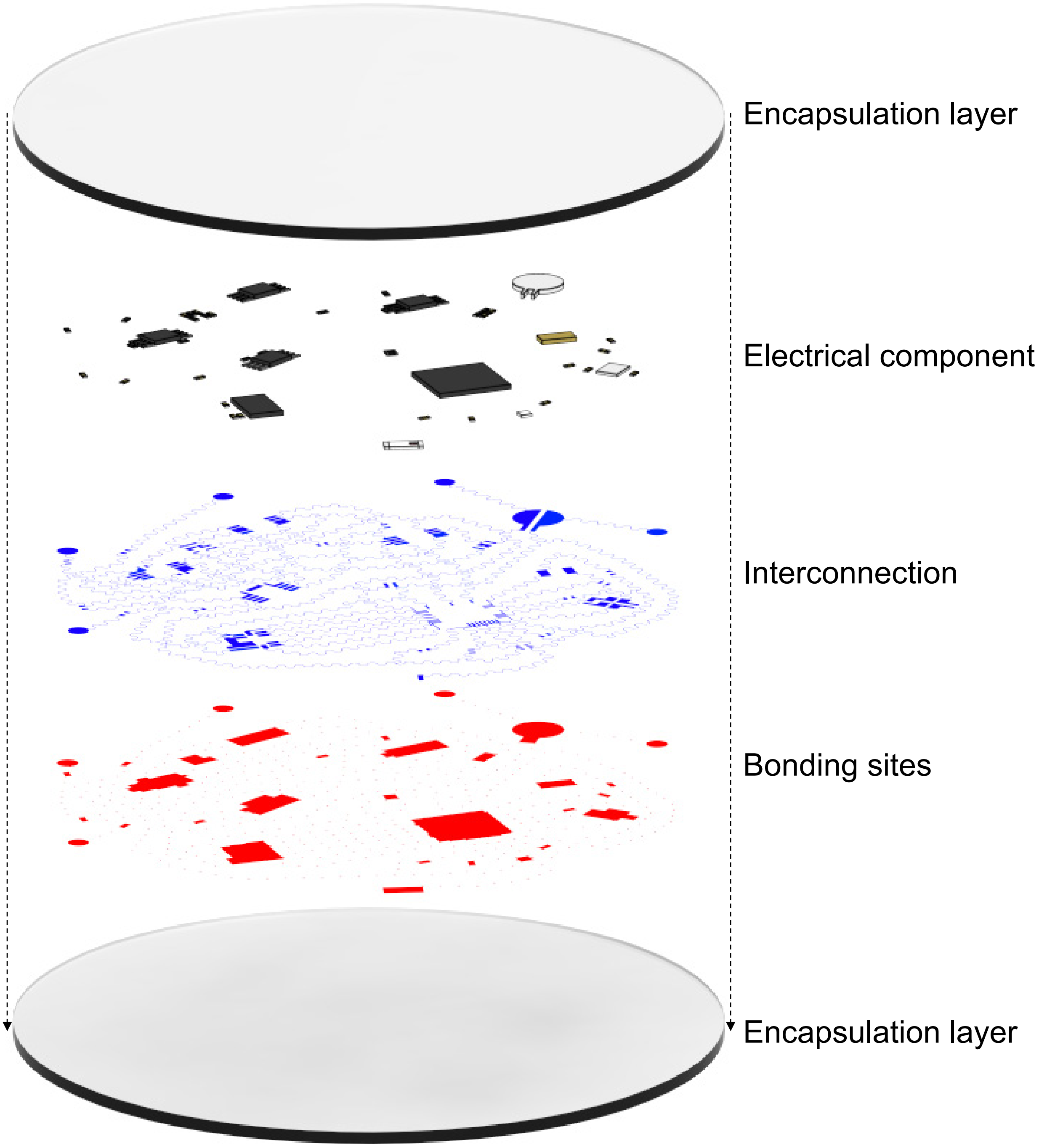

Supplementary Figure 16. Schematics of material integration for 3D coil based circuit

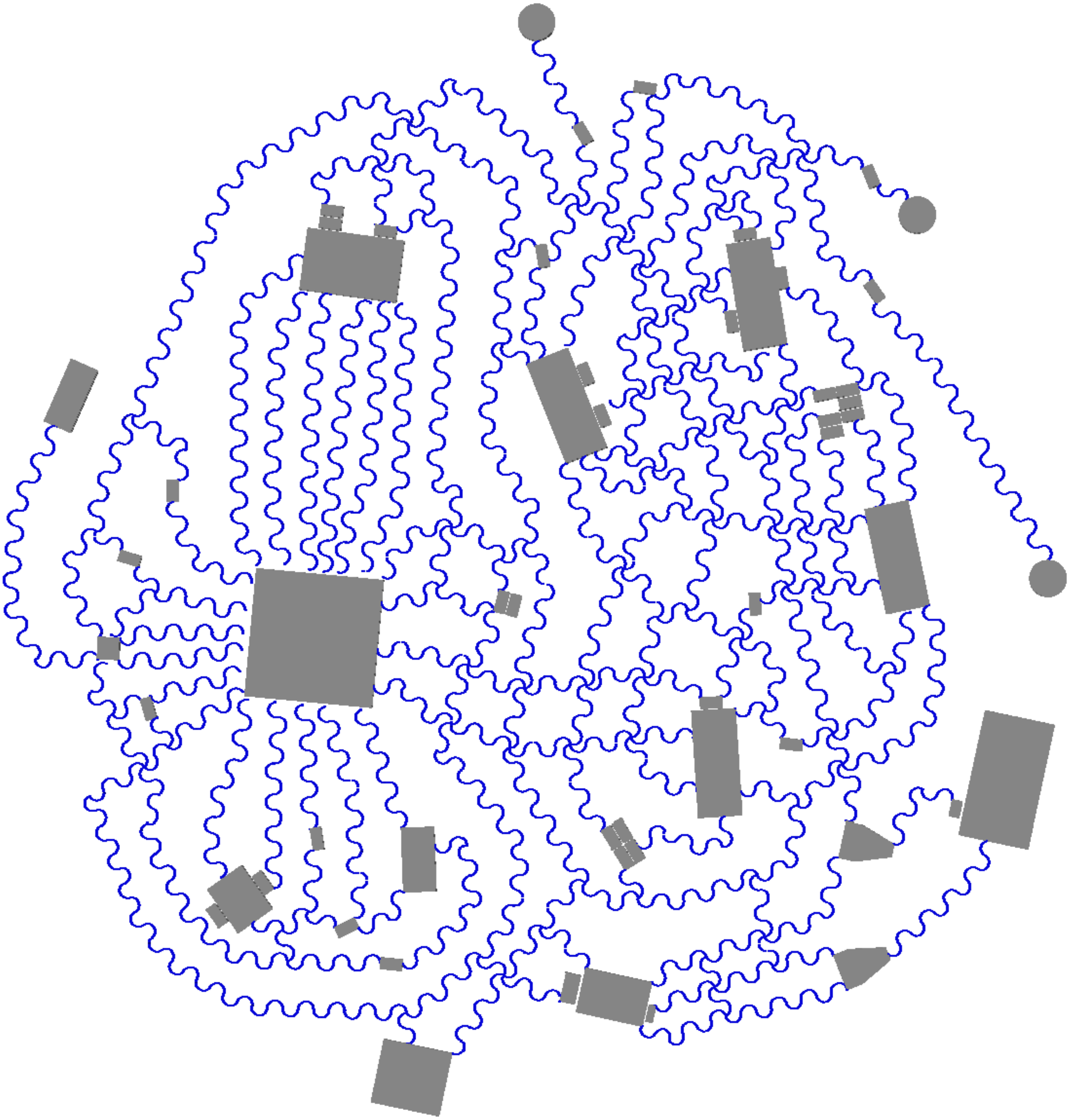

Supplementary Figure 17. Schematic illustration of the 2D precursor for the electronic device system constructed with helical interconnect networks.

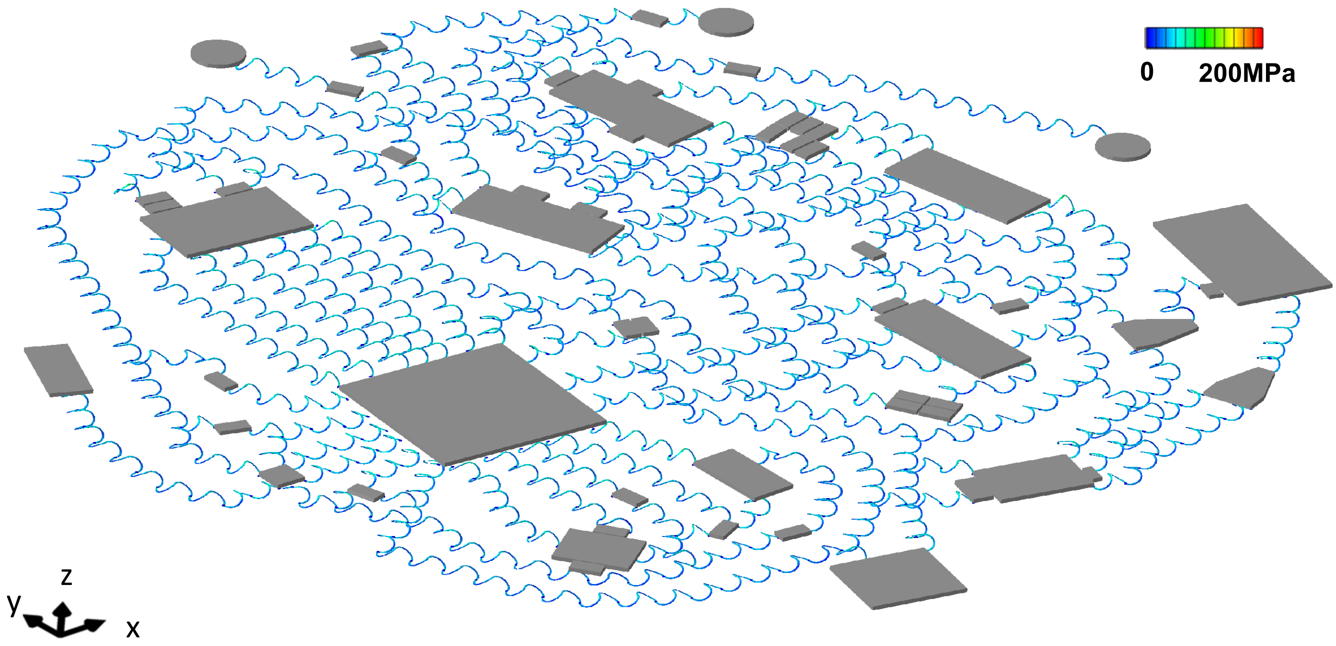

Supplementary Figure 18. FEA prediction on the 3D configuration of the electronic device system constructed with 3D helical interconnect networks. The 3D perspective is the same as the optical image in **Figure 2a**. The color represents the magnitude of Mises stress in the metal layer.

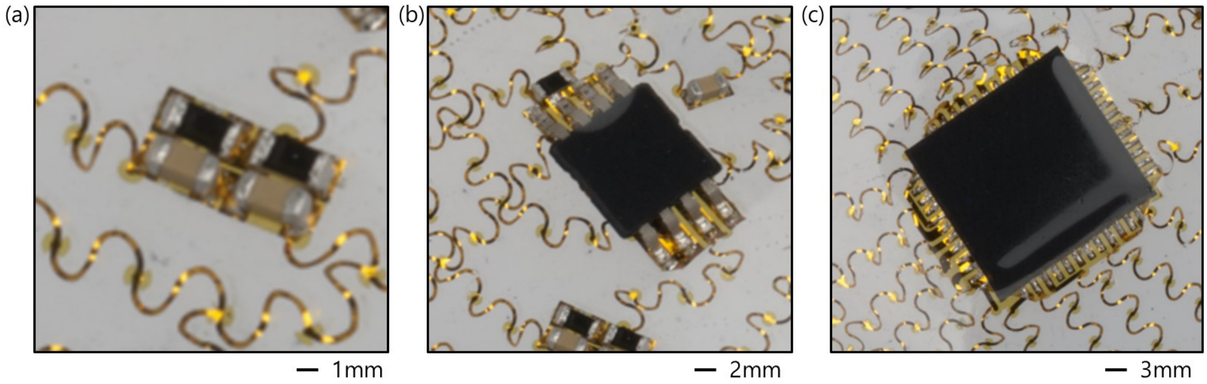

Supplementary Figure 19. Potential delamination at the chip/substrate interface after release of prestrain: (a) fully laminated, (b) partially delaminated, to a slight degree, (c) partially delaminated to a large degree. Even for the case of slight delamination, the electrical interconnection remains stable.

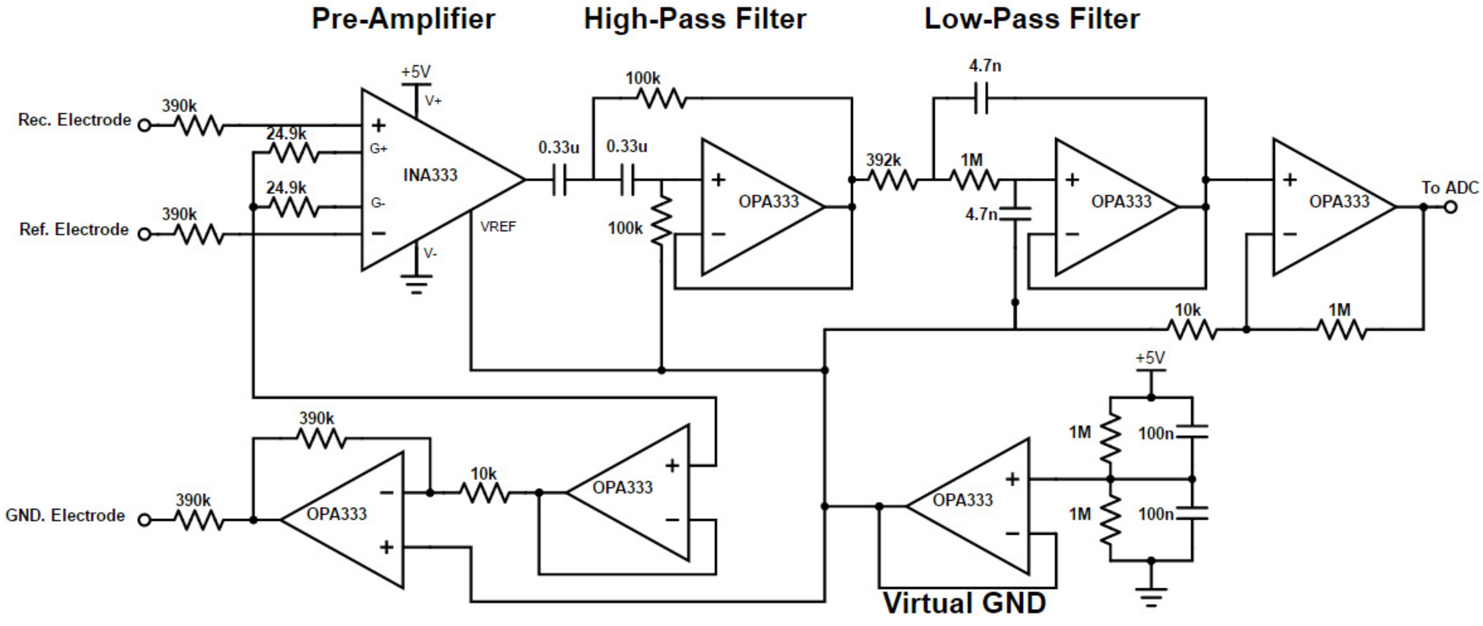

Supplementary Figure 20. Schematic of EP signal measuring Circuit

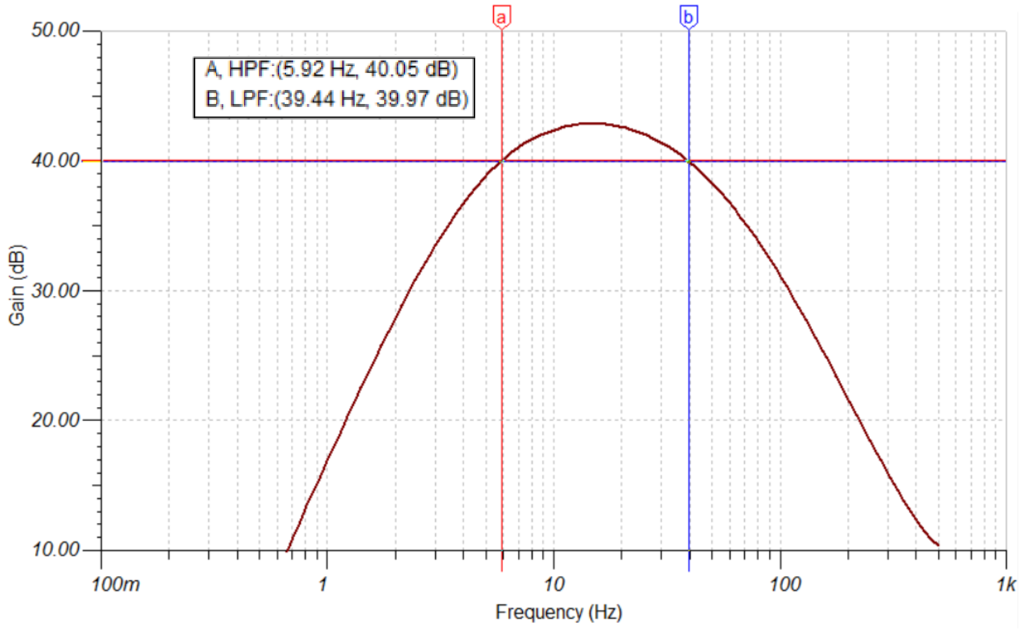

(a) ECG and EEG

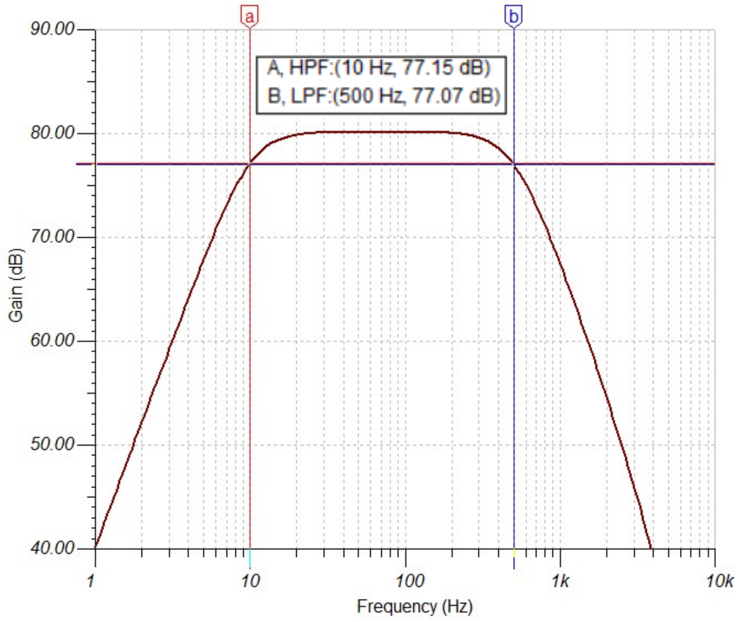

(b) EMG

Supplementary Figure 21. Frequency response of EP circuit

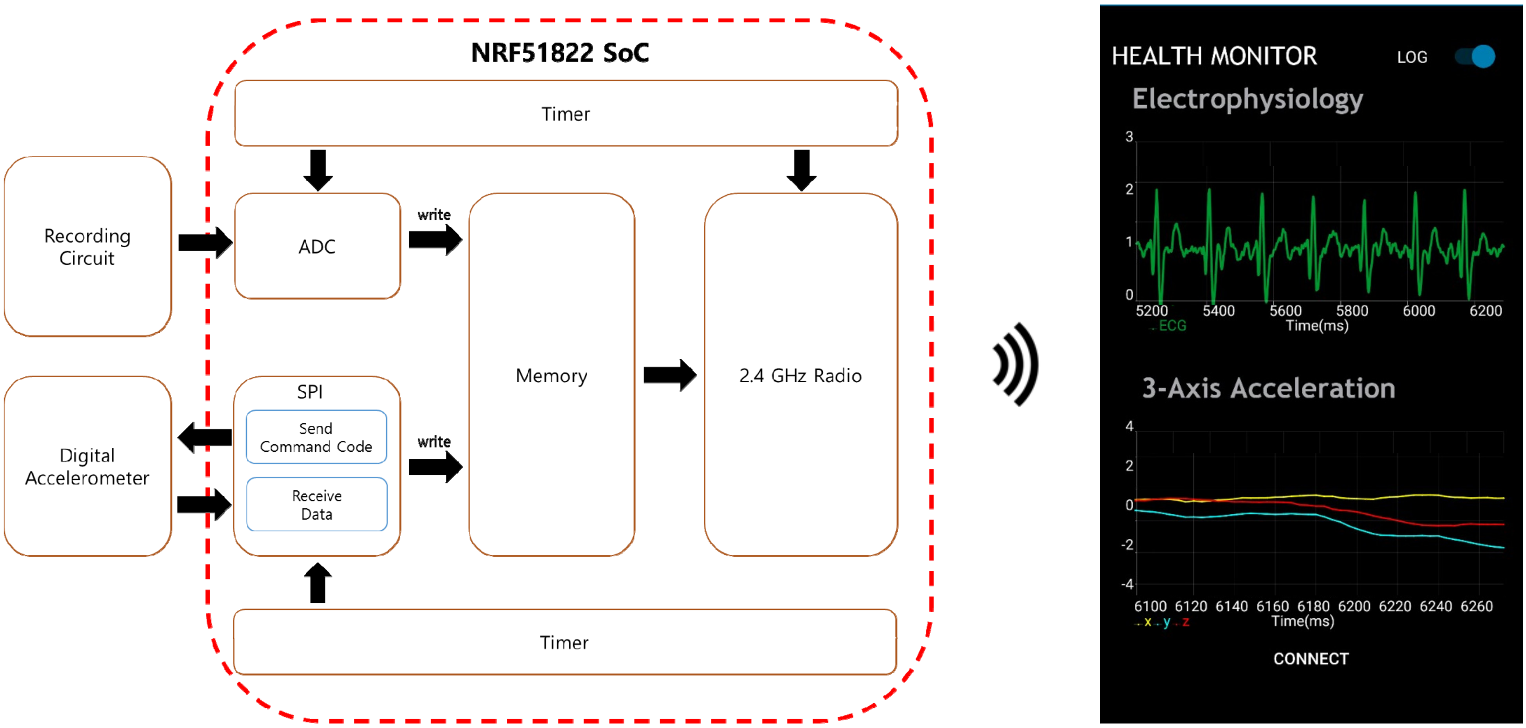

Supplementary Figure 22. Wireless power transfer receiver circuit

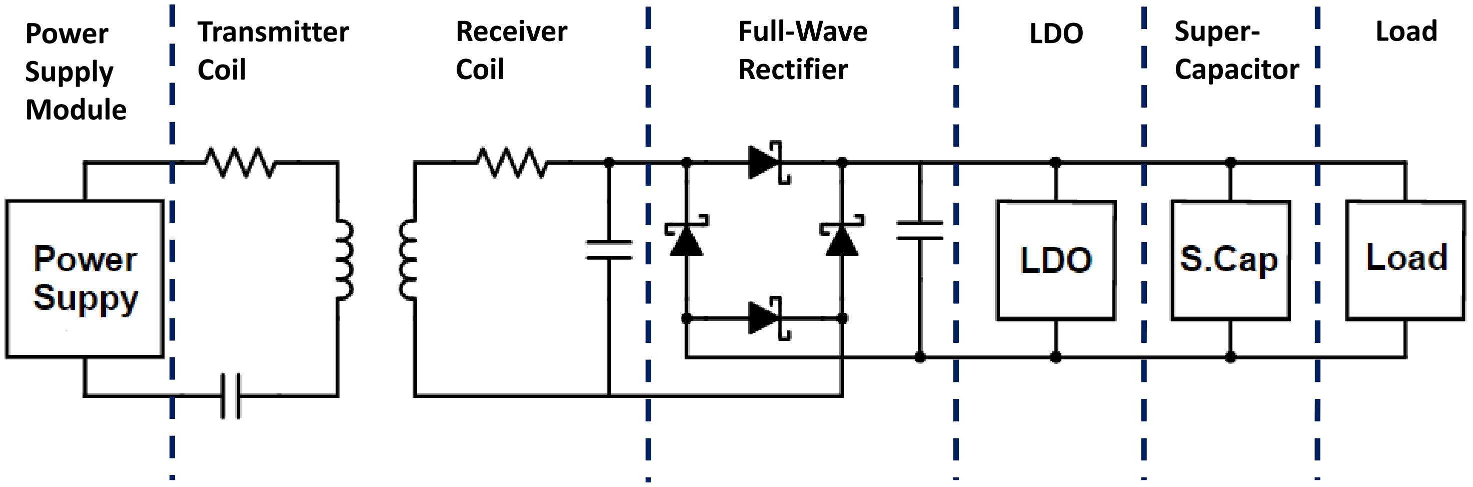

Supplementary Figure 23. Wireless power transfer receiver circuit

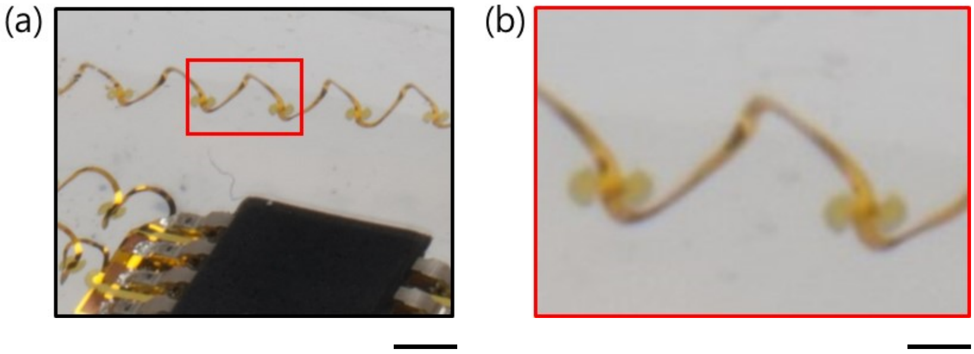

Supplementary Figure 24. Optical images of 3D coil after encapsulation with ultra-soft elastomeric materials (Silbione 4717 A/B, Bluestar Silicones). The 3D structure keeps their original structures well. Scale bars of (a) and (b) are 1mm and 100um, respectively.

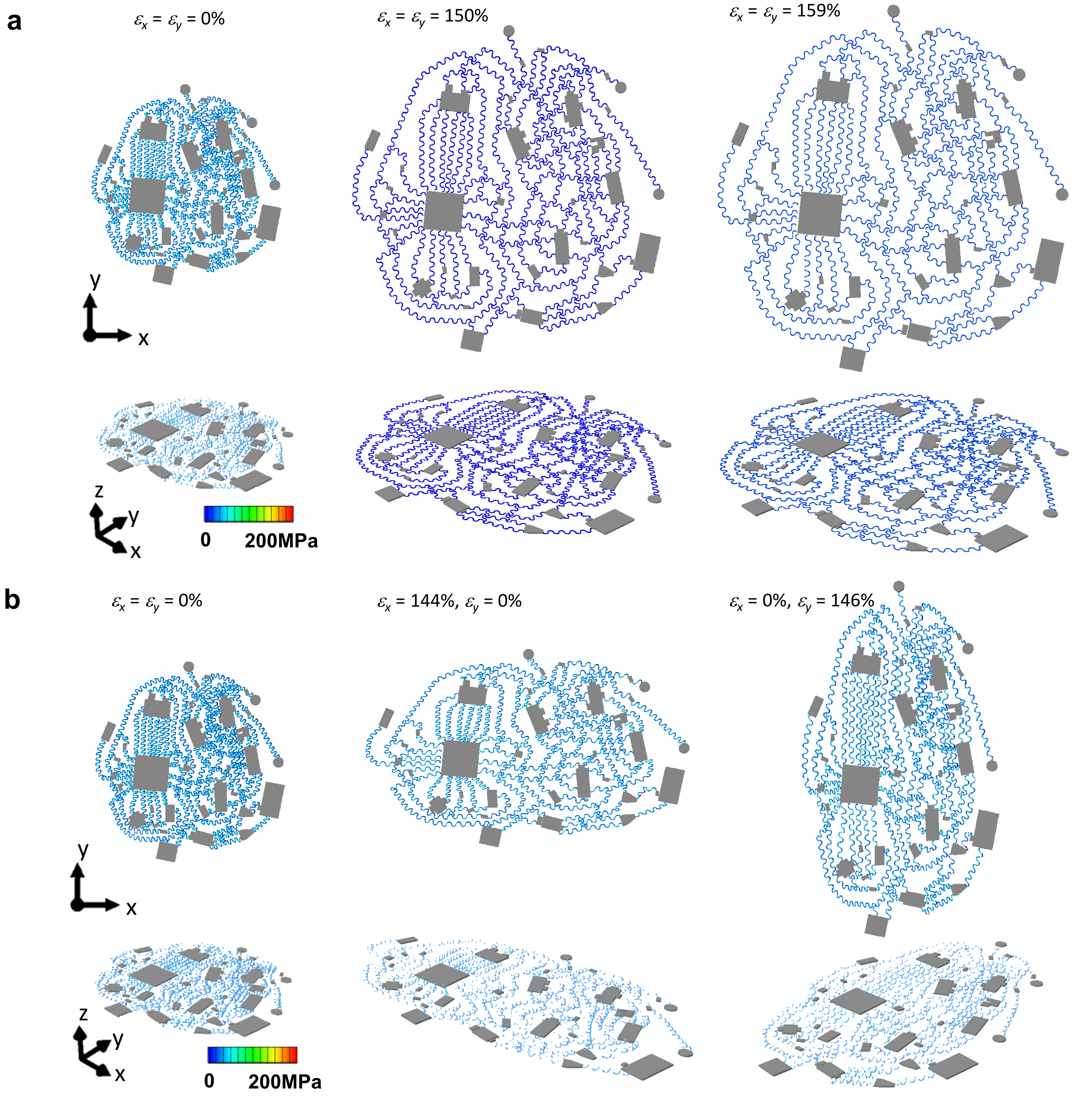

Supplementary Figure 25. Computational studies of the mechanics in an unencapsulated electronic device system with 3D helical interconnect networks. (**a**) System-level FEA results for the undeformed and deformed configurations of the electronic device under equal biaxial stretching. Two levels (150% and 159%) of applied strain are adopted, corresponding to the prestrain used in the 3D assembly and the elastic stretchability of device system. (**b**) System-level FEA results for the undeformed and deformed configurations of the electronic device when uniaxially stretched to the elastic limit (144% for X-axis and 146% for Y-axis). Images with two different view perspectives are shown. The color represents the magnitude of Mises stress in the metal layer.

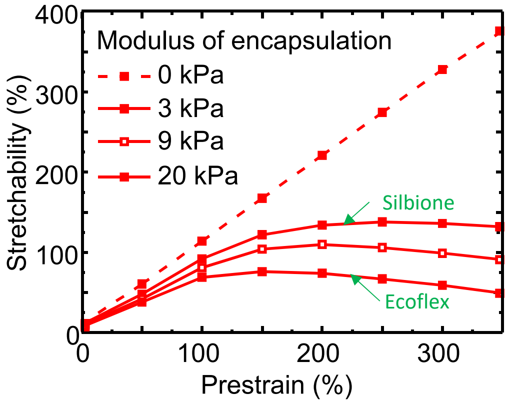

Supplementary Figure 26. Effect of the modulus of the encapsulation material on the elastic stretchability of 3D helical interconnects. Elastic stretchability versus the prestrain for materials with a range of modulus values (from 0 to 20 kPa). The parameters adopted in the simulations are (ϑ_0=180°, w=50 μm, t_{metal}=0.6 μm, t_{PI}=6.0 μm, $E_{substrate}$=20 kPa, E_{metal}=200 GPa).

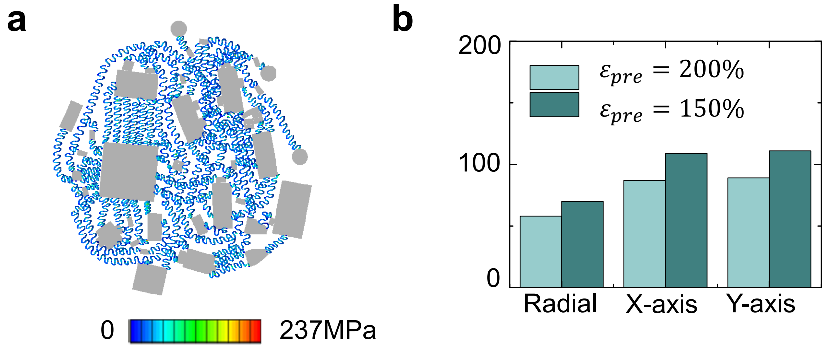

Supplementary Figure 27. Design of circuit system to increase the areal density. (a) FEA result for the deformed configuration of a system with prestrain ε_{pre} (200%). (b) Uniaxial and radial elastic stretchability for two prestrains (150% and 200%), and the same encapsulation strain (30%). The color in (a) denotes the Mises stress.

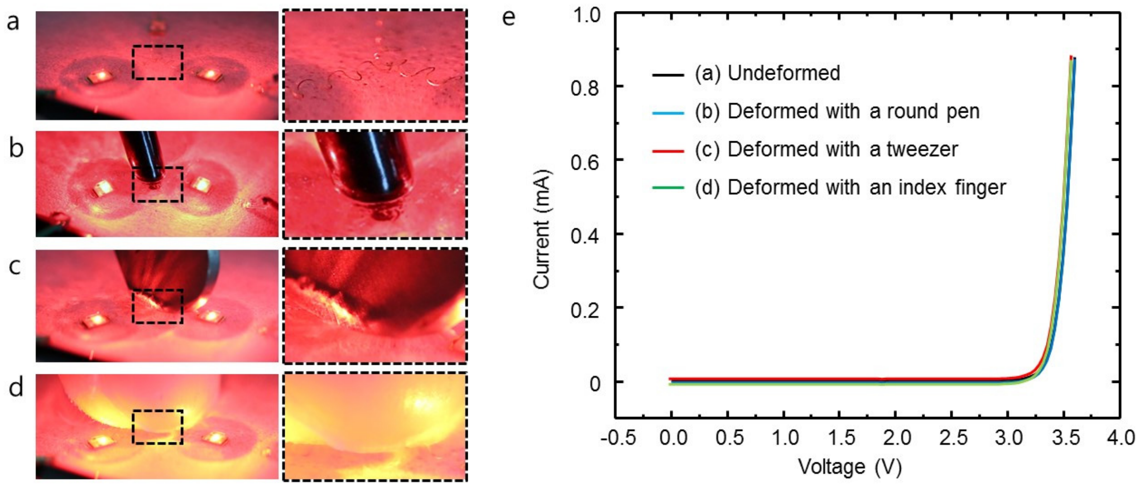

Supplementary Figure 28. A stretchable test structure consisting of a pair of LEDs electrically connected by a 3D helical coil encapsulated in and supported by an elastomer (a). Compressive loads applied with the tip of a pen (b), the end of a pair of tweezers (c) and an index finger (d). The I-V curves under different conditions are in (e).

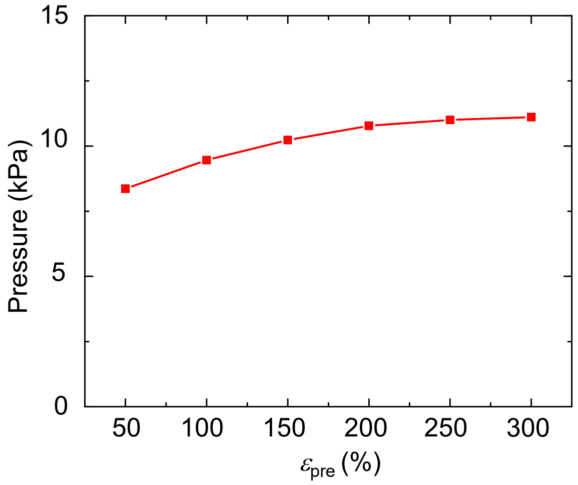

Supplementary Figure 29. FEA results of the pressure needed to compress the 3D helical interconnects such that the out-of-plane dimensions reach 30% of the original values as a function of prestrain used to form the coils.

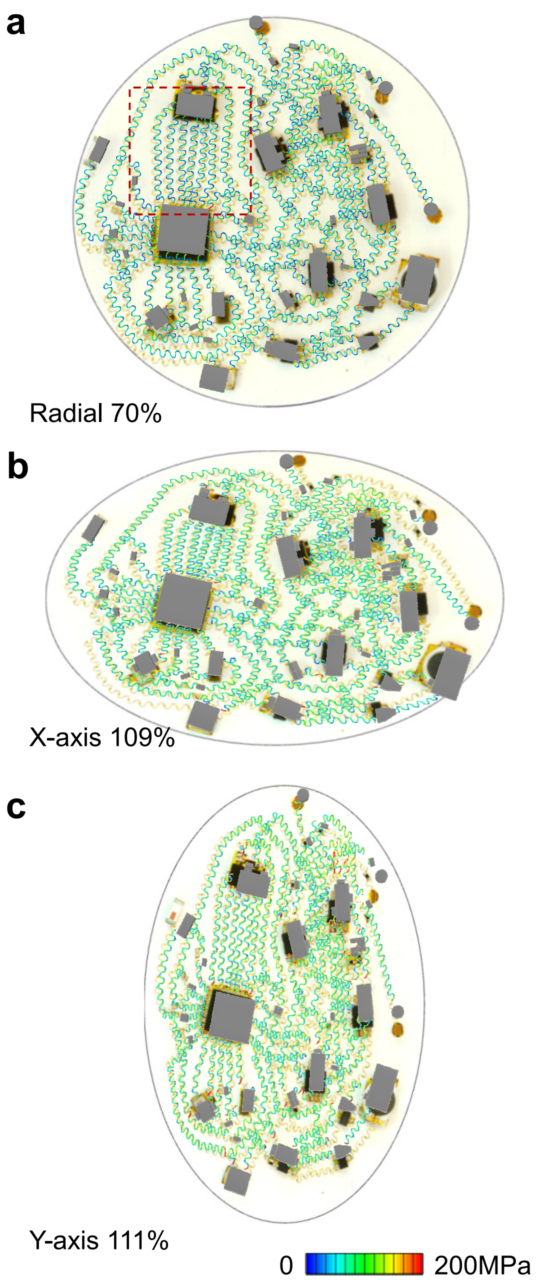

Supplementary Figure 30. Comparison of experiment images and FEA results for the electronic device system with 3D helical interconnect networks under stretching. Optical images overlaid by FEA results for the electronic device system under (a) radial stretching, and uniaxial stretching along (b) X-axis and (c) Y-axis.

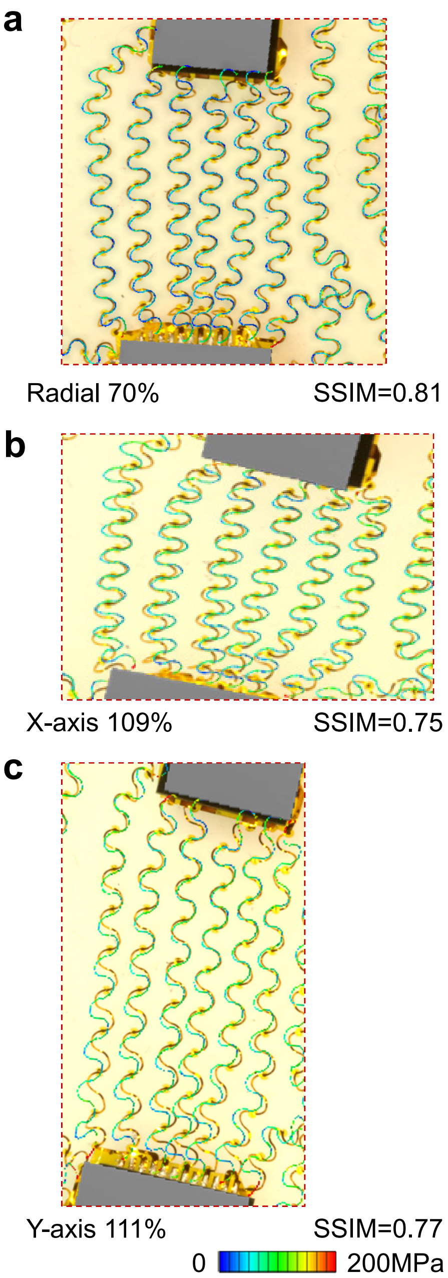

Supplementary Figure 31. Comparison of experiment images and FEA results for a local area of the electronic device system, with results on Structure Similarity (SSIM). Optical image of a local area of the device (marked by red in Supplementary Fig. 24) overlaid by FEA results for the electronic device system under (a) radial stretching, and uniaxial stretching along (b) X-axis and (c) Y-axis. The SSIM index is calculated using $SSIM(x, y) = \left(\frac{s_{xy}+C_3}{s_x s_y+C_3}\right)^{\gamma}$, where s_x and s_y are variance of x and y, and s_{xy} is the covariance of x and y; $C_3 = \frac{1}{2}(k_2 L)^2$ is a variable to stabilize the division with weak denominator, in which k_2 is by default taken as 0.03, and $L = 255$ is the dynamic range of the pixel value for the images; γ=0.5 is a weight used in the calculation.

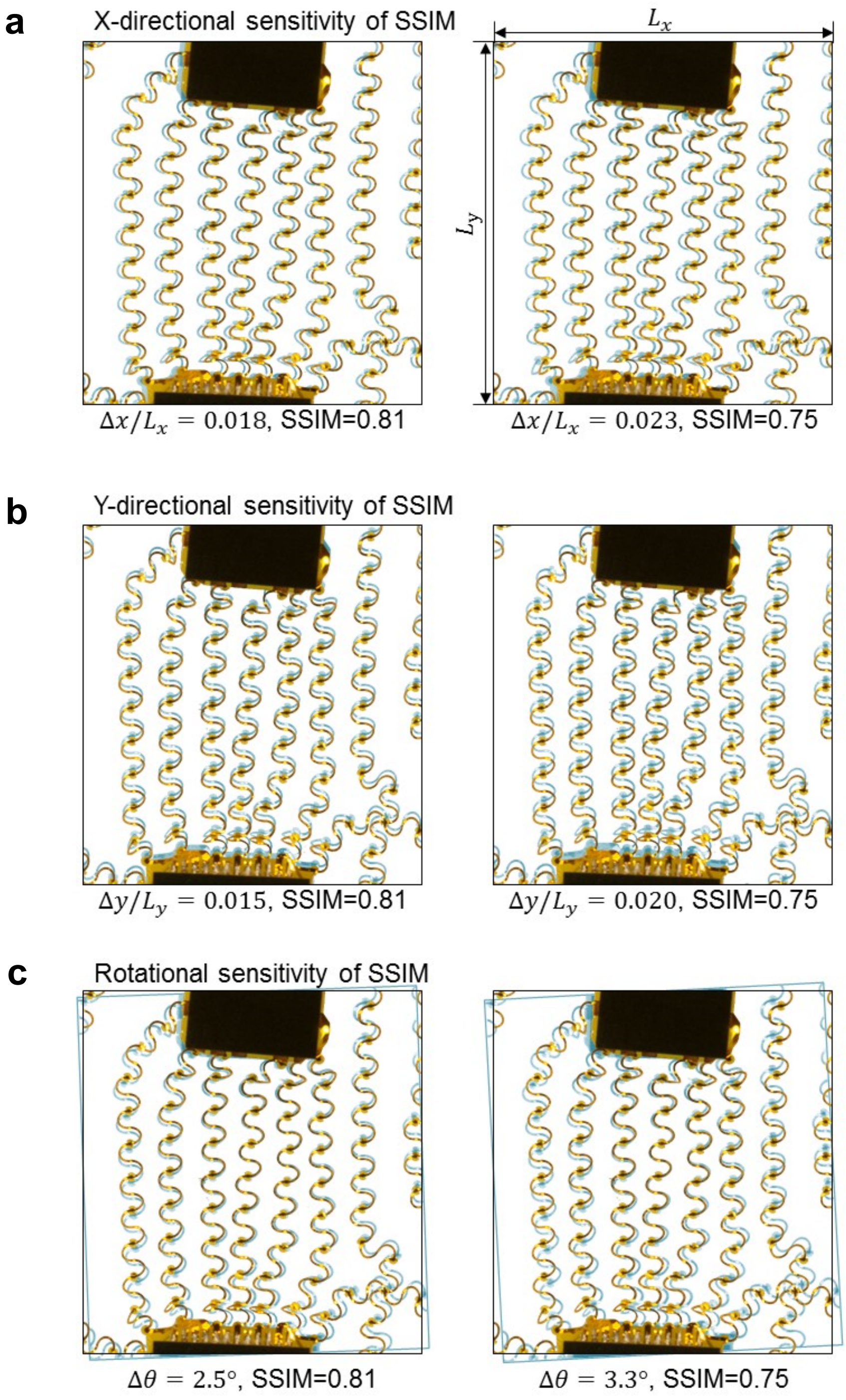

Supplementary Figure 32. Sensitivity of Structure Similarity (SSIM). (a) Optical image of a local area of the device, overlaid by the same image with two different levels of offset along X-axis to yield SSIM of 0.81 and 0.75. (b) Similar results with two different levels of offset along Y-axis. (c) Similar results with two different levels of rotation with regard to the center.

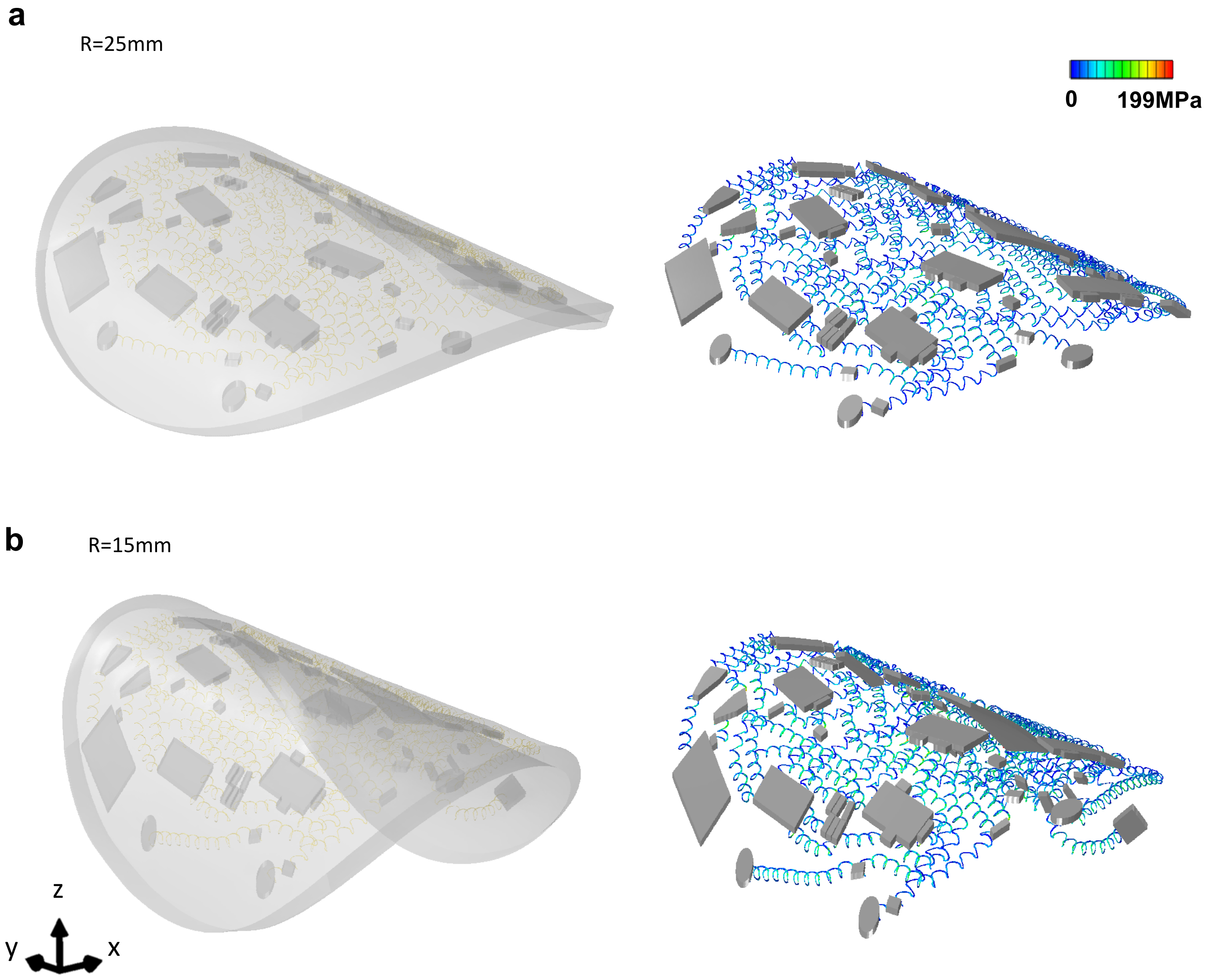

Supplementary Figure 33. Encapsulated electronic device system with 3D helical interconnect networks under bending deformations. (**a**) System-level FEA result for the deformed configuration of the encapsulated electronic device when wrapped onto a rigid cylinder (radius 25 mm). The right image features the distribution of Mises stress in the metal layer. (**b**) Similar results for the encapsulated electronic device wrapped onto a rigid cylinder (radius 15 mm).

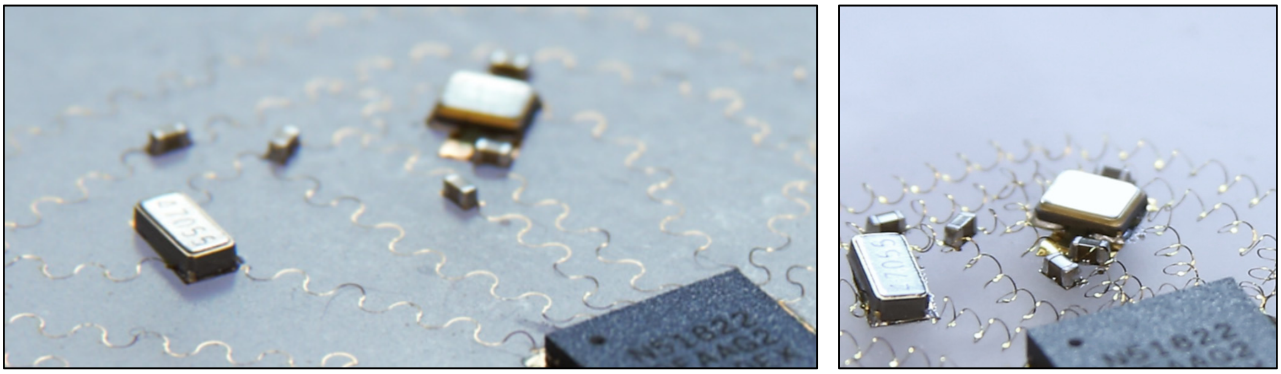

Supplementary Figure 34. Magnified view of the 3D coil based circuit: (a) before and (b) after strain release

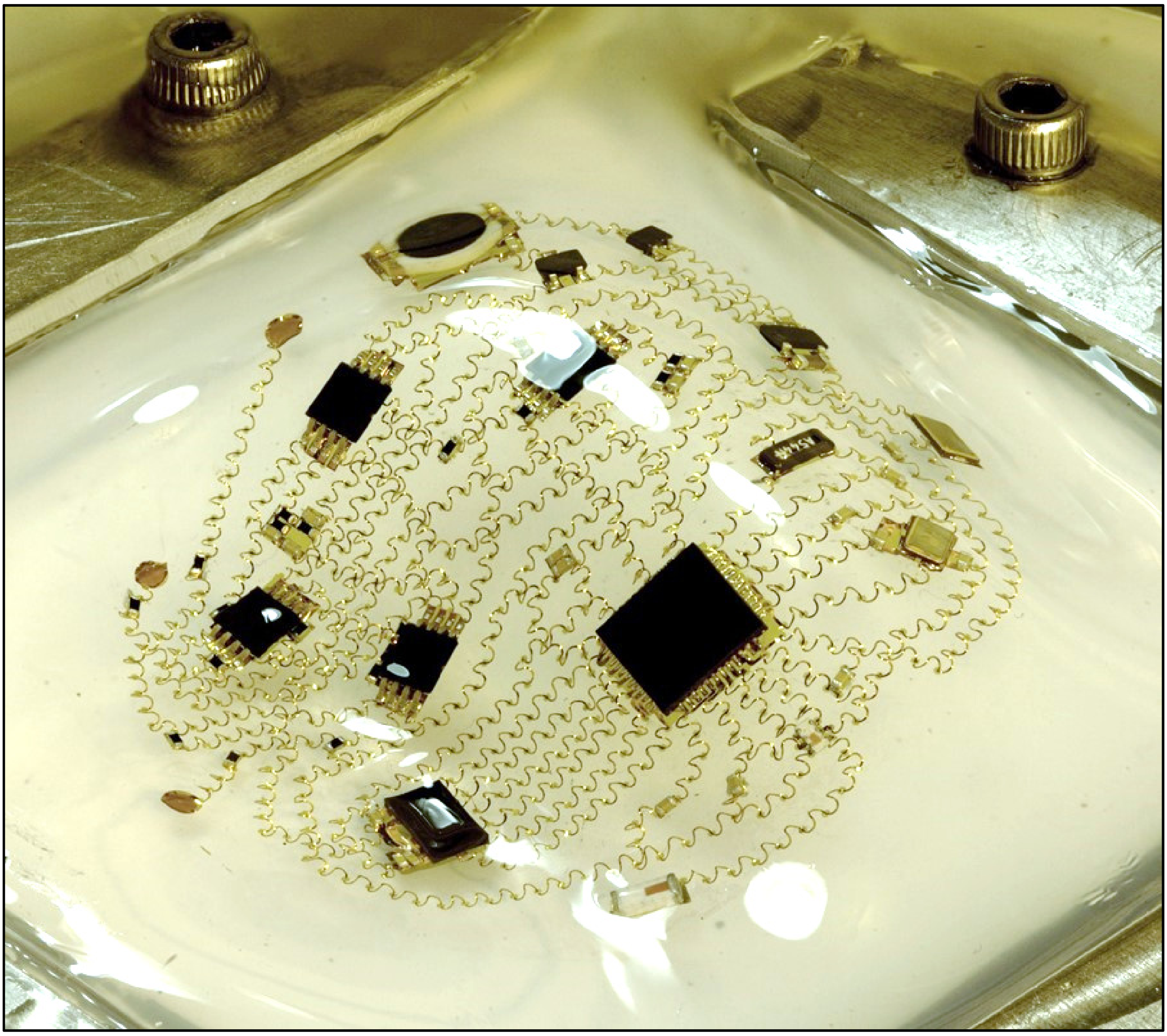

Supplementary Figure 35. Freely deformed 3D coil based circuit

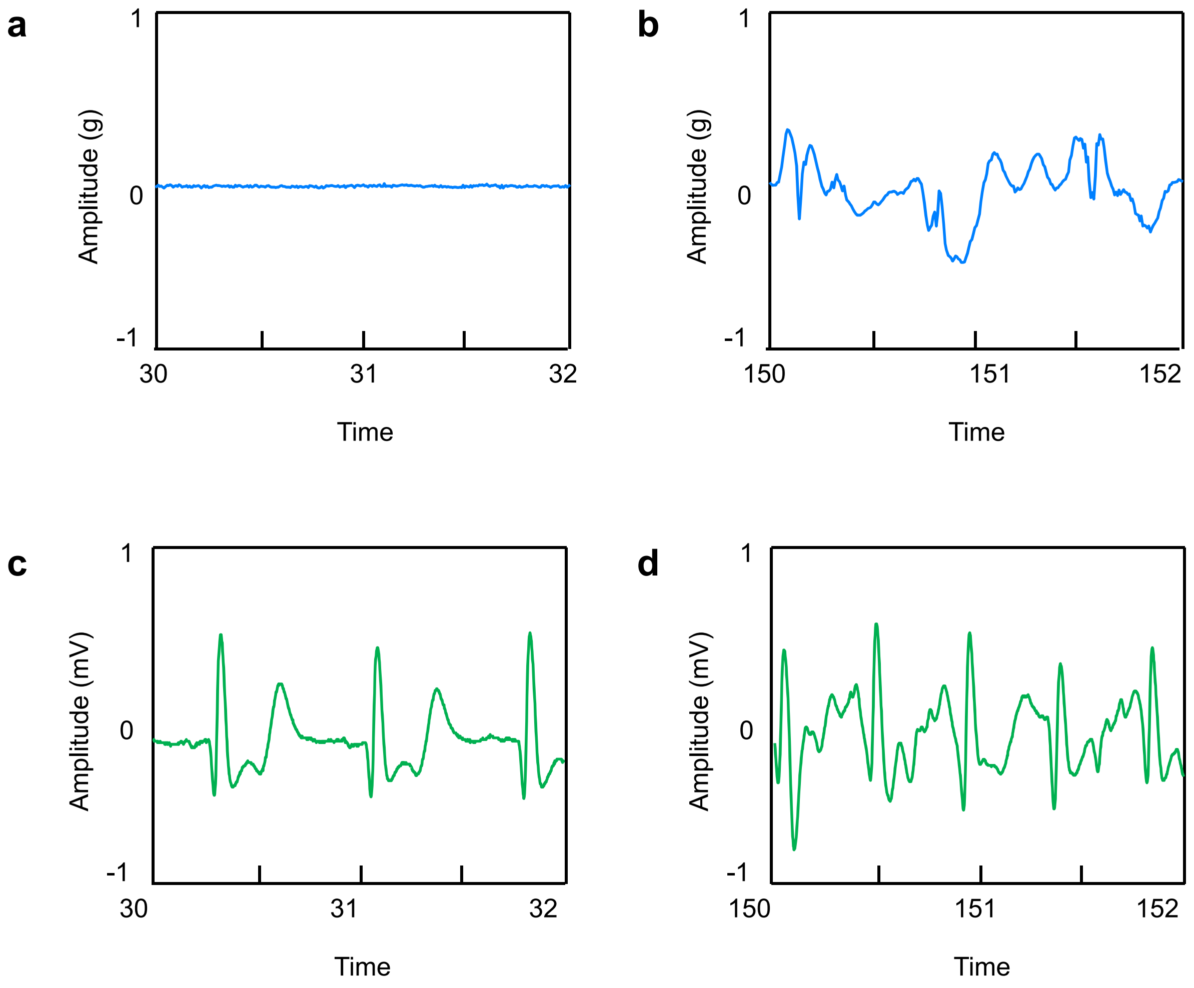

Supplementary Figure 36. Wireless, multimodal monitoring of body activity, through simultaneous measurements: movements of the chest by accelerometry during (a) standing, (b) running, ECG from the chest by electrophysiology electrode during (c) standing, (b) rubbing.

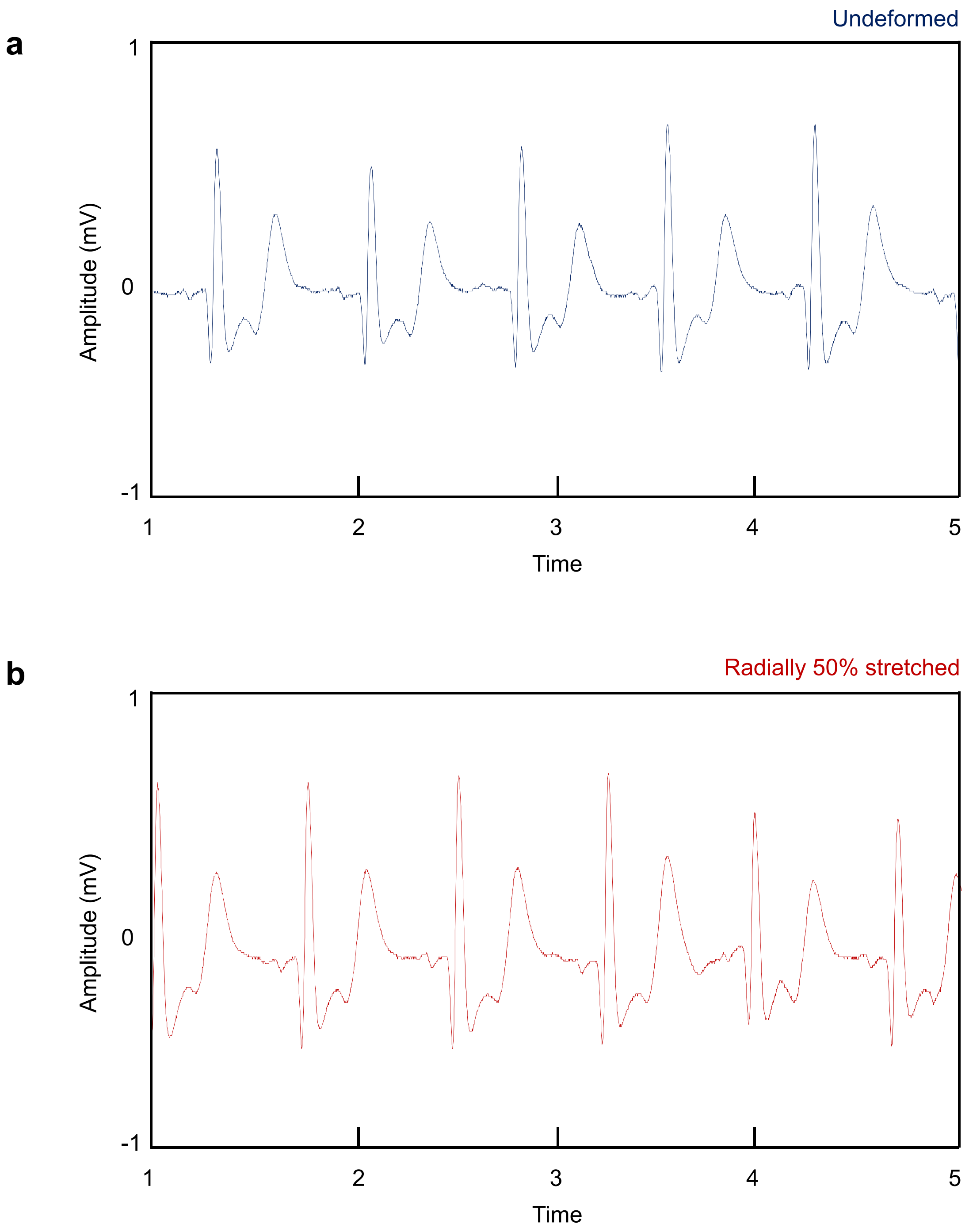

Supplementary Figure 37. Collected ECG signal from the chest: (a) undeformed and (b) radially 50% stretched states

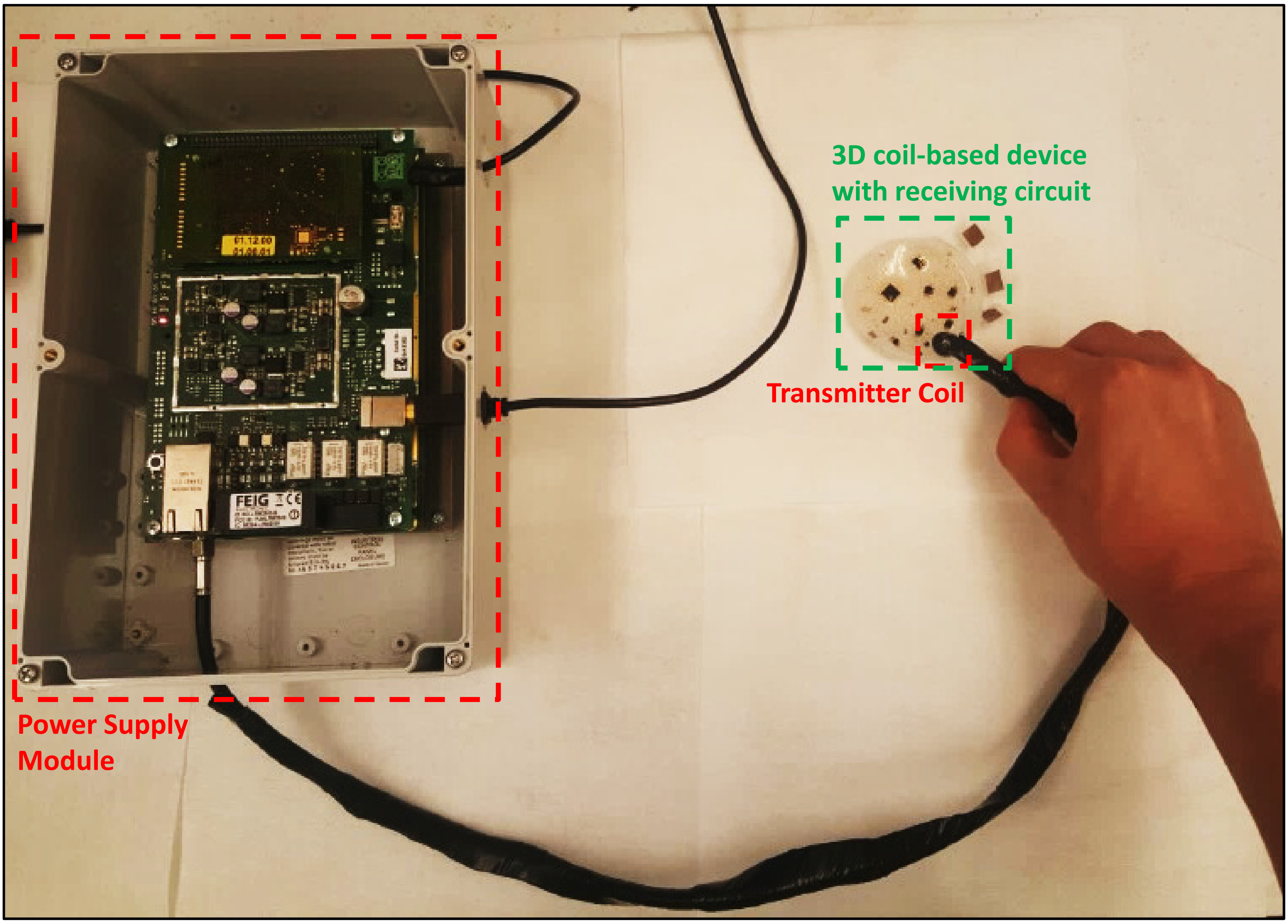

Supplementary Figure 38. Experimental setup for wireless power transfer
